# Human Induced Hepatic Lineage-Oriented Stem Cells: Autonomous Specification of Human iPS Cells toward Hepatocyte-Like Cells without Any Exogenous Differentiation Factors

**DOI:** 10.1371/journal.pone.0123193

**Published:** 2015-04-13

**Authors:** Tetsuya Ishikawa, Momoko Kobayashi, Satoshi Yanagi, Chika Kato, Ryokichi Takashima, Eiji Kobayashi, Keitaro Hagiwara, Takahiro Ochiya

**Affiliations:** 1 Core Facilities for Research and Innovative Medicine, National Cancer Center Research Institute, Chuo-ku, Tokyo, Japan; 2 Takara Bio, Inc., Otsu, Shiga, Japan; 3 Division of Molecular and Cellular Medicine, National Cancer Center Research Institute, Chuo-ku, Tokyo, Japan; University of Kansas Medical Center, UNITED STATES

## Abstract

Preparing targeted cells for medical applications from human induced pluripotent stem cells (hiPSCs) using growth factors, compounds, or gene transfer has been challenging. Here, we report that human induced hepatic lineage-oriented stem cells (hiHSCs) were generated and expanded as a new type of hiPSC under non-typical coculture with feeder cells in a chemically defined hiPSC medium at a very high density. Self-renewing hiHSCs expressed markers of both human embryonic stem cells (hESCs) and hepatocytes. Those cells were highly expandable, markedly enhancing gene expression of serum hepatic proteins and cytochrome P450 enzymes with the omission of FGF-2 from an undefined hiPSC medium. The hepatic specification of hiHSCs was not attributable to the genetic and epigenetic backgrounds of the starting cells, as they were established from distinct donors and different types of cells. Approximately 90% of hiHSCs autonomously differentiated to hepatocyte-like cells, even in a defined minimum medium without any of the exogenous growth factors necessary for hepatic specification. After 12 days of this culture, the differentiated cells significantly enhanced gene expression of serum hepatic proteins (*ALB*, *SERPINA1*, *TTR*, *TF*, *FABP1*, *FGG*, *AGT*, *RBP4*, and *AHSG*), conjugating enzymes (*UGT2B4*, *UGT2B7*, *UGT2B10*, *GSTA2*, and *GSTA5*), transporters (*SULT2A1*, *SLC13A5*, and *SLCO2B1*), and urea cycle-related enzymes (*ARG1* and *CPS1*). In addition, the hepatocyte-like cells performed key functions of urea synthesis, albumin secretion, glycogen storage, indocyanine green uptake, and low-density lipoprotein uptake. The autonomous hepatic specification of hiHSCs was due to their culture conditions (coculture with feeder cells in a defined hiPSC medium at a very high density) in self-renewal rather than in differentiation. These results suggest the feasibility of preparing large quantities of hepatocytes as a convenient and inexpensive hiPSC differentiation. Our study also suggests the necessity of optimizing culture conditions to generate other specific lineage-oriented hiPSCs, allowing for a very simple differentiation.

## Introduction

The forced expression of four transcription factor genes, *Oct3/4*, *Sox2*, *Klf4*, and *c-Myc*, in mouse embryonic and adult fibroblast induces pluripotent stem cells (iPSCs) [1]. It is noteworthy that the four defined factors, as well as other factors, also generate iPSCs from human somatic cells [2–6]. In addition, it was shown that *OCT3/4*, *SOX2*, and *KLF4* without *c-MYC* could generate iPSCs [7]. Human iPSCs (hiPSCs) are similar to human embryonic stem cells (hESCs) [8] in terms of the ability to self-renew in vitro and differentiate into cells of all three germ layers. Therefore, the use of hiPSCs could lead to significant changes in the future of medicine [9].

Hepatocytes would be promising targeted cells for hiPSCs in medical applications, as the availability of human hepatocytes is often limited by shortages of donor organs. They are expected to be an option for regenerative medicine [10], drug discovery, and studies on etiology [11]. Hepatocytes generated from individual donors with each variation might mimic individual differences in drug metabolism, which often cause unpredictable side effects. Moreover, human leukocyte antigen (HLA)-matching hepatocytes might make it possible to omit immunosuppressive drugs in cell-based therapies for liver diseases, including hepatitis, cirrhosis, and hepatocellular carcinoma.

Differentiation of hiPSCs to a specific cell lineage is an essential step for the application. It was suggested that donor differences are an important determinant of the propensity for hepatic differentiation of various hiPSC lines [12]. Nevertheless, multistep hepatic differentiation protocols have been reported and applied to hiPSCs. The preparation has required several growth factors [13–16], compounds [17], or gene transfer [18]. Hepatocyte-like cells have thus far been prepared using growth factors and compounds by a typical multistep protocol of differentiation culture [19].

Here, we report that human induced hepatic lineage-oriented stem cells (hiHSCs) can be established as a new type of hiPSC with only some modifications of their culture conditions in both generation and self-renewal. They are expanded under non-typical coculture with feeder cells in mTeSR1 medium on gelatin-coated dishes at a very high density. The potential hepatic specification of hiHSCs is defined by their expression profiles of hepatic markers under a self-renewal culture.

## Materials and Methods

### Human Adult Gastric Tissue-Derived Cells

The gastric tissues were derived from pathologically defined non-cancerous parts of the stomach of a patient with stomach cancer as surgical waste after an operation under informed consent. The tissue did not include intestinal tissue embryologically closer to liver cells. Gastric cells were prepared as follows. The tissues were washed with Hank’s balanced salt solution (HBSS, Invitrogen) and minced with scissors into pieces. The pieces were further washed with HBSS until a supernatant became clear. After removal of the supernatant, Dulbecco's modified Eagle medium (DMEM, Gibco) supplemented with 0.1% collagenase (Wako Pure Chemical) and 1X antibiotic/antimycotic (Gibco) was added to the tissue precipitates, and stirring was performed at 37°C for 1 hr with a shaker. After confirming that the precipitated tissues had been fully digested, DMEM supplemented with 10% fetal bovine serum (FBS) was added, and the suspension was then centrifuged. After removal of the supernatant, DMEM supplemented with 10% FBS was added, and the suspension was centrifuged again. Cells were finally suspended in DMEM supplemented with 1X antibiotic/antimycotic and 10% FBS.

### Generation of hiHSCs

Human adult (43 years old, white, female) dermal fibroblasts were obtained from a company (Lonza). The cells were seeded with FGM-2 BulletKit medium (Lonza) in Matrigel-coated dishes. One day later, the cells at approximately 5–10% confluency were incubated with the pantropic retrovirus vector solution at 37°C for 24 hr in a 5% CO_2_ incubator. The replication-incompetent pantropic Moloney murine leukemia virus (MMLV)-derived retrovirus vector pMX solution was used for the ectopic expression of human *OCT3/4*, *SOX2*, and *KLF4*. Following gene transduction, the culture was replaced with mitomycin C-treated mouse embryonic fibroblast (MEF; Millipore)-conditioned media every three days until the fibroblasts reached 100% confluency at a very high density. The confluent culture was further refreshed with mTeSR1 medium (STEMCELL Technologies) daily from days 18 to 27, the MEF-conditioned medium daily from days 28 to 33, and mTeSR1 medium daily from days 34 to 38. Clone AFB1-1 was isolated at day 39 after the three-gene transduction using forceps under a microscope.

The gastric cells (from a Japanese male, 67 years old) were seeded on collagen-coated dishes with Dulbecco's modified Eagle medium supplemented with 10% fetal bovine serum. One day later, the cells at approximately 5–10% confluency were incubated with the pantropic retrovirus vector solution at 37°C for one day. The MEFs were seeded following the infection. The culture was replaced with the MEF-conditioned medium every three days until proliferating cells reached 100% confluency at a very high density. The confluent culture was further refreshed with mTeSR1 medium every day from day 31. Clones NGC1-1 and NGC1-2 were isolated at days 41 and 46, respectively.

### Subculture of hiHSCs

The isolated clones were subcultured in each well of gelatin-coated 24-well plates. After an expansion culture, each clone was further cultured to each well of gelatin-coated 6-well plates and finally cultured in a gelatin-coated 100-mm dish. The expanded clones AFB1-1, NGC1-1, and NGC1-2 were treated with a dissociation solution (0.25% trypsin—EDTA [Gibco] and 1% collagenase [Invitrogen]) or 0.25% trypsin—EDTA and passaged in mTeSR1 supplemented with 10–20 μM Y-27632 (Calbiochem and Wako) to avoid cell death. Those clones were cultured with the MEFs (5 X 10^4^ cells/cm^2^) mainly in mTeSR1 medium and occasionally in Primate ESC medium (ReproCell) on gelatin-coated dishes. Total RNA was prepared from each 100-mm dish and subjected to microarray analyses. The established clones continued to expand under coculture with the MEFs mainly in mTeSR1 medium and occasionally in ReproStem (ReproCell) medium on gelatin-coated dishes. Although mTeSR1 has been supplied as a feeder-free defined medium, it was applied with the MEFs. ReproStem and Primate ESC were used as conventional media for an hESC culture with the MEFs.

As a self-renewal subculture for the following robust differentiation, the clones AFB1-1 and NGC1-1 were passaged with treatment with trypsinization and 10 μM Y-27632. The clones were seeded as single cells or very small clusters at a density of 1–2 X 10^6^ cells per 100-mm dish when the passages were split at 1:12 to 1:30. The clones were expanded under coculture with the MEFs (2.5–4.2 X 10^4^ cells/cm^2^) in mTeSR1 medium on gelatin-coated dishes. The medium was usually refreshed only at days 1, 4, and 6, and the self-renewing clones became almost 100% confluent at a very high density of approximately 1.2–3.0 X 10^7^ cells per 100-mm dish (2–5 X 10^5^/cm^2^) at day 7. Such a subculture continued to be repeated.

### Differentiation Culture in ReproStem Medium without FGF-2

Clone AFB1-1 or NGC1-1 was trypsinized and then seeded with mTeSR1 medium without Y-27632 at a density of 1 X 10^6^ cells per well in Matrigel-coated 6-well plates. The seeded cells were cultured for approximately 5 hr and replaced with ReproStem medium without FGF-2. ReproStem medium consists of undefined components for an hESC/hiPSC culture. The medium was further refreshed at days 4 and 8, and the differentiation culture continued until day 12. Total RNA was prepared from each well and then subjected to real-time reverse transcription-polymerase chain reaction (RT-PCR).

### Differentiation Culture in Essential 6 Medium

Clone AFB1-1 was trypsinized and then seeded with mTeSR1 medium without Y-27632 at a density of 1 X 10^6^ cells per well in Matrigel-coated 6-well plates. The seeded cells were cultured for approximately 5 hr and replaced with E6 medium (Life Technologies). The medium was further refreshed at day 6 or at days 4 and 8. The differentiation culture continued until day 12 and then was subjected to further analyses.

### Pantropic Retrovirus Vectors

The study was approved by the Institutional Recombinant DNA Advisory Committee. Recombinant vector plasmids were constructed from the replication-incompetent Moloney murine leukemia virus-derived retrovirus vector plasmid pMXs (kindly provided by Dr. Kitamura) and plasmids carrying human *OCT3/4*, *SOX2*, and *KLF4* genes (Open BioSystems). Pantropic recombinant retroviruses were prepared by transfecting vector plasmids (5 μg of *OCT3/4*-pMXs, 2.5 μg of *KLF4*-pMXs, 1.25 μg of *SOX2*-pMXs, and 5 μg of VSV-G-pCMV [Cell Biolabs]) with 45 μL of FuGENE HD (Roche) to the confluent Plat-GP packaging cells (kindly provided by Dr. Kitamura) in a 100-mm dish, followed by incubation in DMEM supplemented with 10% fetal bovine serum. From 48 hr after transfection, the supernatant from the Plat-GP culture was collected several times at intervals of approximately 12 hr and passed through a 0.45 μm pore filter. The resulting retrovirus vectors were used for the ectopic expression of human *OCT3/4*, *SOX2*, and *KLF4* genes.

### MEF-Conditioned Medium

hESC medium was prepared as DMEM/F12 supplemented with 10% Knockout Serum Replacement (KSR, Invitrogen), 2 mM GlutaMAX (Invitrogen), 1×nonessential amino acids (Sigma), 1×antibiotic antimycotic solution, and 10 ng/mL bFGF (Peprotech). Mitomycin C-treated MEFs were seeded at approximately 1.5 X 10^6^ cells/100-mm dish. The medium was conditioned with the MEFs (Millipore and ReproCell) for one day, and the MEF-conditioned medium was collected several times and supplemented with 0.1 mM 2-mercaptoethanol (Sigma), 10 ng/mL bFGF, and an additional 10% KSR (final conc. 20% KSR) just before use.

### Passage

Cells were washed with a phosphate-buffered saline solution, detached with trypsin/EDTA (0.25% trypsin/1 mM EDTA in phosphate-buffered saline [PBS], Gibco) or a dissociation solution (0.25% trypsin, 0.2% collagenase type IV [Invitrogen], 1 mM calcium chloride, and 20% KSR) at 37°C for 3 to 5 min, and suspended with DMEM/F12. After centrifugation, the cells were seeded and cultured for one day in mTeSR1 or ReproStem medium with 10 μM ROCK inhibitor Y-27632 (Calbiochem and Wako).

### Microarray Analysis

Using the AllPrep DNA/RNA Mini Kit (Qiagen), total RNA was prepared from a hepatocellular carcinoma cell line (HuH-7) and uncultured human adult hepatocytes (obtained from the Health Science Research Resources Bank) as well as clones AFB1-1, NGC1-1, and NGC1-2 that were cultured on the MEFs (5 X 10^4^ cells/cm^2^) with mTeSR1 (StemCell Technologies) medium in gelatin-coated 100-mm dishes before long-term serial passage. The microarray study was carried out using a Whole Human Genome Oligo Microarray 4X44K (Agilent). The analysis was performed by Bio Matrix Research according to Agilent technical protocols. Data from these experiments and the GEO database for hESCs (hES_ES01, GSM194392; hES_BG03, GSM194391; hES_H9, GSM194390) and hiPSCs (201B7, GSM241846) established from human adult fibroblasts (fibroblast, GSM242095) were analyzed with GeneSpring GX 11.5 software (Agilent). The microarray data reported in this paper are available on the Gene Expression Omnibus under accession number GSE63844.

### G Banding and Multicolor FISH Analyses

The three established clones were intensively expanded under coculture with the MEFs on gelatin mainly in mTeSR1 medium and occasionally in ReproStem medium. After long-term serial passage, karyotyping and chromosome abnormalities of hiHSCs were tested by Giemsa banding (G banding) and multicolor FISH analyses. Each clone was pretreated with 0.02 μg/mL colecemid (Sigma) overnight, incubated with 0.075 M KCl and 1% citric acid for 40 min, and then fixed with Carnoy’s fixative. For G banding, cells were stained with Giemsa and analyzed by microscopy. For multicolor FISH analyses, cells were hybridized with the multicolor FISH probe (Vysis) and analyzed using a DMRA2 fluorescence microscope (Leica) by Nihon Gene Research Laboratories.

### Fluorescent Immunocytochemistry

Cells were fixed with 10% formaldehyde in PBS for 15 min. For intracellular antigens, cells were permeabilized with 0.1% Triton X-100/PBS before blocking. After being washed with PBS, the cells were incubated with a blocking solution/PBS (Nacalai Tesque) at room temperature for 1 hr. The cells were incubated overnight at 4°C with primary antibodies in a 10% blocking solution/PBS. After being washed with PBS, the cells were further incubated with fluorescent-conjugated secondary antibodies (Molecular Probes) for 2 hr at room temperature in a 10% blocking solution/PBS. After washing, fluorescence was imaged using a Leica DMI 6000B Inverted Fluorescent Microscope, DFC360FX camera, and AF6000 microscope imaging software; a Nikon ECLIPSE TE300 Inverted Fluorescent Microscope, Digital Sight DS-U2 microscope camera controller, and NIS-Elements microscope imaging software; or a Keyence All-in-One Fluorescent Microscope BZ-9000. The manufacturers and concentrations of primary and secondary antibodies and isotype controls are listed in S9 and S10 Tables, respectively.

### Alkaline Phosphatase Staining

Alkaline phosphatase staining was performed using a TRACP and ALP Double-Stain Kit according to the manufacturer’s instructions (Takara Bio).

### HLA Genotyping

According to HLA Laboratory instructions, alleles at the human leukocyte antigen HLA-A, -B, -Cw, and -DRB1 loci were identified by the Luminex microbeads method and platform (100 System: Luminex) as follows: DNA sampling was carried out by amplifying the target genes by PCR using biotin labeled primers. Amplified fragments were hybridized with sequence-specific oligonucleotide probes conjugated to color-coded microbeads. Hybridization was identified by cytometry dual-laser analysis.

### STR Analysis

According to the manufacturer’s instructions (Promega), the GenePrint 10 System was used for the analysis of 10 loci consisting of TH01, TPOX, vWA, Amelogenin, CSF1PO, D16S539, D7S820, D13S317, D5S818, and D21S11 present within the human genome.

### Real-Time RT-PCR Gene Expression Analysis

Total RNA was prepared using the miRNeasy Mini Kit (Qiagen). One microgram total RNA was used for reverse transcription, which was carried out using a PrimeScript RT Master Mix (Takara Bio). Quantitative PCR was carried out with SYBR Premix Ex Taq II (Tli RNaseH Plus) (Takara Bio) using the CFX96 Real-Time PCR Detection System (Bio-Rad). PCR primer sets are listed in S8 Table. The PCR data were digitized and analyzed using CFX Manager Software (Bio-Rad). Data from the RNA of clone AFB1-1 or the RNA of human adult and fetal livers (Clontech Laboratories) were used as a relative standard, and a calibration curve was drawn. The expression of genes of interest was normalized to that of *GAPDH* in all samples. Data are presented as mean+SEM and represent a minimum of three independent samples with at least two technical duplicates.

### ELISAs

Human-specific albumin (ALB) and alpha-1-fetoprotein (AFP) were measured by enzyme-linked immunosorbent assays (ELISAs) according to the manufacturer’s instructions (Takara Bio). Data are presented as mean±SEM and represent a minimum of three independent samples with at least two technical duplicates.

### Urea Production

A urea nitrogen detection kit was purchased from Kanto Chemicals, and urea production was measured by an automatic analyzer, BM8060 (JEOL), according to SRL’s laboratory manual. Data are presented as mean+SEM and represent a minimum of three independent samples.

### PAS Staining

A Periodic Acid-Schiff (PAS) staining kit was purchased from Muto Pure Chemicals. At day 12 of the differentiation culture in Essential 6 (E6) medium, PAS staining was performed according to the manufacturer’s instructions. The staining was then examined by microscopy.

### Cellular Uptake of ICG

Indocyanine Green (ICG) (Tokyo Chemical Industry) was dissolved in DMSO at 100 mg/mL and then diluted with DMEM supplemented with 10% FBS at a ratio of 1:200. The medium including ICG was prepared at 37°C at a final concentration of 0.5 mg/mL. At day 12 of the differentiation culture, E6 medium was replaced by the medium including ICG, and then hepatocyte-like cells were incubated in a 5% CO2 incubator at 37°C for 5 hr. The medium was discarded, and the cells were washed with PBS. The cellular uptake of ICG was then examined by microscopy.

### Cellular Uptake of LDL

BODIPY FL—labeled low-density lipoprotein (LDL) (Molecular Probes) at 1 mg/mL was diluted with E6 medium at a ratio of 1:100. The medium including LDL was prepared at a final concentration of 10 μg/mL. At day 12 of the hepatic differentiation, the culture was washed with PBS and replaced by the medium including LDL, and then hepatocyte-like cells were incubated in a 5% CO2 incubator at 37°C for 24 hr. The supernatant was discarded, and the culture was washed with PBS and replaced again by the medium including LDL. Hepatocyte-like cells were further incubated for 24 hr. The cellular uptake of BODIPY FL—labeled LDL was then examined by fluorescent microscopy.

### Flow Cytometry Analysis

The hepatic differentiation culture of clone AFB1-1 continued until day 12 with E6 medium on Matrigel, and then the resultant hepatocyte-like cells were subjected to flow cytometry analyses. Primary cultured human fetal hepatocytes and uncultured human adult hepatocytes were also analyzed as controls. Uncultured human adult hepatocytes were obtained through the Health Science Research Resources Bank, and human fetal hepatocytes were purchased from Cell Systems. For the hepatocyte-like cells and the fetal hepatocytes, single-cell suspensions were obtained by dissociating the culture with 0.25% trypsin-1 mM EDTA at 37°C for 3–5 min. The uncultured human adult hepatocytes have been prepared from tissues by collagenase and stocked in liquid nitrogen. After washing with DMEM, cells were fixed with 4.0% paraformaldehyde (Wako) at RT for 15 min and then washed three times with PBS. The fixed cells were treated with a blocking solution/PBS (Nacalai Tesque) containing 10% FBS at 4°C for 1 hr. Cells were treated by gently shaking with mouse monoclonal antibodies against asialoglycoprotein receptor 2 (ASGPR2/hepatocyte-specific antigen, Abcam), glial fibrillary acid protein (GFAP/isotype control/IgG2b, IBL), and an unknown antigen (negative control/IgG1/MOPC-21, BioLegend) in a 20% blocking solution/PBS containing 2% FBS at 4°C overnight and then washed with PBS. Cell staining was performed with PE-labeled goat anti-mouse secondary antibodies (BioLegend) in a 20% blocking solution/PBS containing 2% FBS at 4°C for 2 hr. The cells were washed with PBS and then suspended with a 10% blocking solution in PBS. The stained cells were analyzed with the MACSQuant analyzer (Miltenyi Biotec), and the data were digitized using MACSQuantify (Miltenyi Biotec). Anti-GFAP monoclonal antibodies were used as an isotype control (IgG2b). Mouse IgG1/MOPC-21 was quality-control tested by immunofluorescent staining with flow cytometry analysis as a negative control. Data are presented as the mean and represent a minimum of three independent samples. The manufacturer and concentrations of primary and secondary antibodies and isotype controls are listed in S9 and S10 Tables, respectively.

### Transplantation of Self-Renewing hiHSCs into NOD/SCID Mice

As a self-renewal subculture before transplantation, clone AFB1-1 was routinely passaged with treatment of trypsinization and 10 μM Y-27632. The trypsinized cells were seeded as single cells or very small clusters at a density of 1.0–3.0 X 10^6^ cells per gelatin-coated 100-mm dish when the passages were split at a ratio of 1:10 to 1:30. The cells were self-renewed under coculture with MEFs (2.5–4.0 X 10^4^ cells/cm^2^) in mTeSR1 medium on gelatin-coated dishes. The medium was refreshed at days 1, 4, and 6, and the self-renewing hiHSCs became 100% confluent at a very high density at day 7. Cell suspensions (1.0, 2.0, and 3.7×10^7^ cells/250 μL of Essential 6 medium) were mixed with 250 μL of Matrigel (BD), and the mixtures were subcutaneously injected into the backs of nonobese diabetic/severe combined immunodeficiency (NOD/SCID) mice (Charles River Laboratories Japan) using 1-mL syringes with 18-gage needles. The mice were bled and sacrificed at days 66, 83, and 84 under anesthesia. Teratomas were excised and fixed with 4% paraformaldehyde in PBS and subjected to histological analyses.

### Statistical Evaluation

Statistical analyses were performed by using a standard unpaired Student’s *t* test (two-tailed, 99.99% confidence intervals).

### Ethics Statement

The remnant human tissue specimens were provided under approval of the Institutional Review Board in the Japan Health Sciences Foundation/the Health Science Research Resources Bank and the National Cancer Center of Japan. Written, informed consent from the donor was obtained for the use of these samples in research. The Health Science Research Resources Bank has been transferred to Department of Disease Bioresources Research, Japanese Collection of Research Bioresources, National Institute of Biomedical Innovation (http://bioresource.nibio.go.jp/). The animal experiment was approved by the Institutional Animal Experimentation Ethics Committee.

## Results

### Unique hiPSCs Are Generated by a Modified Protocol

To isolate colonies with clear edges, we modified a protocol that was previously shown to generate hiPSCs [6, 20]. Using the modified protocol (see Materials and Methods), we generated unique hiPSCs from human somatic cells as follows. Adult dermal fibroblasts and gastric tissue-derived cells were infected with pantropic MMLV-derived vectors (pMXs) [21, 22] carrying the three genes *OCT3/4*, *SOX2*, and *KLF4*. After the three-gene transduction, adult dermal fibroblasts were grown in the MEF-conditioned ESC medium, and gastric tissue-derived cells were grown in the conditioned medium under coculture with the MEFs. After reaching confluency of human cells at a very high density, those cells formed colonies with clear edges through the culture in mTeSR1 medium [23]. The colonies were picked up and cocultured with the MEFs mainly in mTeSR1 medium and occasionally in Primate ESC medium on gelatin-coated dishes. Although mTeSR1 has been supplied as a feeder-free defined medium according to the manufacturer’s instruction, the clones were allowed to effectively expand under non-typical coculture with the MEFs in this defined medium. Such hiPSCs formed many small colonies with clear edges, as single cells did not give rise to cell death even after passages with trypsinization when 10 μM Y-27632 was utilized [24]. In contrast, typical hiPSCs (201B7) could not be substantially expanded under non-typical coculture with the MEFs in mTeSR1 medium on gelatin-coated dishes. A clone, which was established from adult fibroblasts, was termed AFB1-1. Two clones, which were established from a non-cancerous gastric cell, were termed NGC1-1 and NGC1-2. Clones AFB1-1, NGC1-1, and NGC1-2 were evidently identical to their respective starting cells in the HLA type (S1 and S2 Tables) and Short Tandem Repeats (STR) genotype (S3 Table).

### hiHSCs Are a New Type of hiPSC That Express Markers of Both hESCs and Hepatocytes

The three established clones, AFB1-1, NGC1-1, and NGC1-2, self-renewed under non-typical coculture with the MEFs in mTeSR1 medium on gelatin-coated dishes and were analyzed by DNA microarray. Hierarchical cluster analysis using the gene set defined by the International Stem Cell Initiative (S4 Table) [25] reveals that the three clones clustered with hESCs (ES01, H9, BG03) and typical hiPSCs (201B7) but separated from fibroblasts, a hepatocellular carcinoma cell line (HuH-7), and an uncultured human adult hepatocyte (S1 Fig). This analysis indicates that the three established clones were very similar to hESCs and hiPSCs in the expression profiling of the gene set. In detail, unlike typical hiPSCs and hESCs, the three established clones expressed the genes of alpha-1-antitrypsin (*SERPINA1*) and alpha-fetoprotein (*AFP*) that are lineage markers for hepatocytes. Gene expression of tyrosine aminotransferase (*TAT*), which is a mature hepatic marker, was prominent only in the adult hepatocyte, not in the established clones. In other aspects, female-derived clone AFB1-1 expressed the *XIST* gene, but male-derived clones NGC1-1 and NGC1-2 did not.

**Fig 1:**
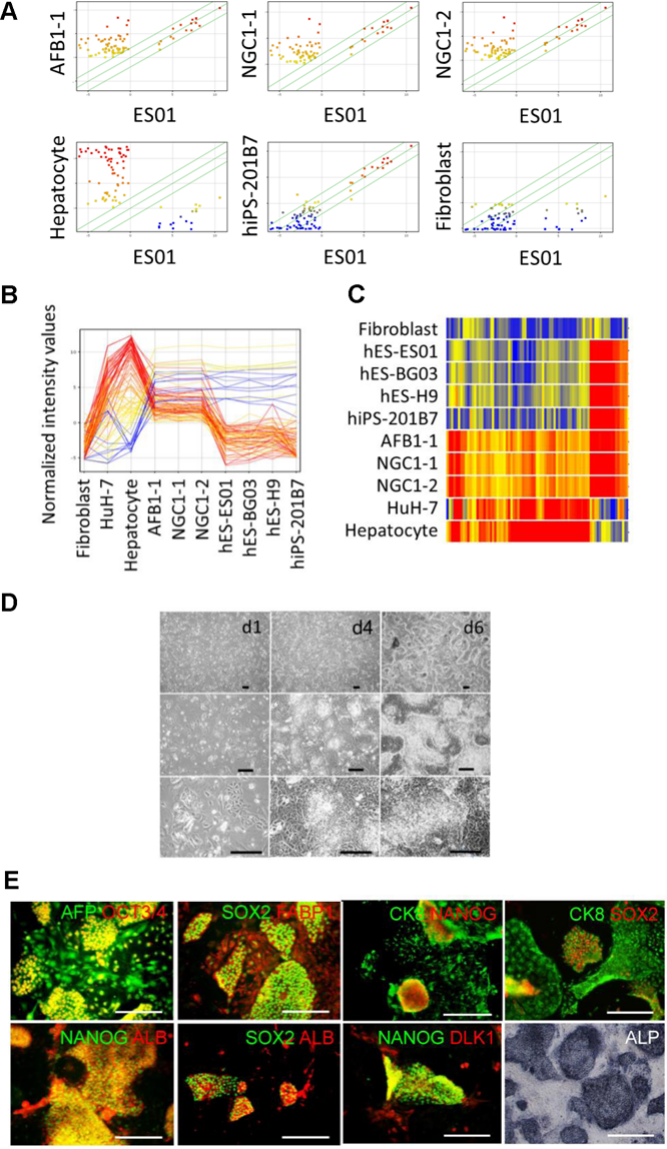
Established hiHSCs express markers of both ESCs and hepatocytes. (**A, B, C**) The gene expression of established hiHSCs was analyzed by DNA microarray. Shown are data characterized by (**A**) scatter plots, (**B**) profiling plots, and (**C**) a heat map of the expression profiles of embryonic stem cell (ESC)- and hepatocyte-enriched genes to compare hiHSCs (clones AFB1-1, NGC1-1, and NGC1-2) with fibroblasts, human ESCs (ES01, BG03, and H9), hiPSCs (201B7), a hepatocellular carcinoma cell line (HuH-7), and human adult hepatocytes. Normalized fluorescent intensity values range from red (high) to blue (low) coloring. Scatter plots are colored by the values of cell samples along the Y-axis. Profiling plots are colored by the values of hepatocytes. All three of the clones exhibit unique gene expression profiles expressing both hESC-enriched genes and hepatocyte-enriched genes distinct from fibroblasts, ESCs, hiPSCs, HuH-7, and hepatocytes. See also S2, S3, and S4 Figs. (**D**) Phase contrast micrographs showing the morphology of hiHSCs (clone AFB1-1) at days 1, 4, and 6 after passage. Scale bar represents 100 μm. See also S5 Fig. (**E**) Staining of immunocytochemistry and alkaline phosphatase (ALP) activity of hiHSCs (clone AFB1-1). Immune double-staining confirms the co-expression of ESC-specific transcription factors (OCT3/4, SOX2, and NANOG) and hepatocyte-marker proteins (AFP, FABP1, ALB, CK8, and DLK1). ALP activity was stained with violet coloring. Scale bar represents 100 μm. See also S7, S8, S9, and S10 Figs.

To investigate the expression of other hepatic genes, we extensively listed the genes (S5 Table) expressing in both the hepatocytes and the three clones except for those in fibroblasts, hESCs (ES01), and hiPSCs (201B7). Using the combined list (S7 Table) of the hepatic genes (S5 Table) and the ESC-enriched genes (S6 Table), we compared the established clones AFB1-1, NGC1-1, and NGC1-2 with hESCs (ES01, BG03, and H9), hiPSCs (201B7), a hepatocellular carcinoma cell line (HuH-7), the hepatocyte, and fibroblasts in scatter plots (Fig 1A), profiling plots (Fig 1B), and a heat map (Fig 1C, S4 Fig) of gene expression. The 50th percentile of fluorescent intensity distribution was normalized across arrays and was treated as the minimum of the expressed genes. Normalized fluorescent intensity values ranged from red (high) to blue (low) coloring. Scatter plots indicate that clones AFB1-1, NGC1-1, and NGC1-2 extensively expressed multiple hepatic genes including highly specific markers, *SERPINA1*, albumin (*ALB*), transthyretin (*TTR*), angiotensinogen (*AGT*), alpha-2-HS-glycoprotein (*AHSG*), fatty acid binding protein 1 (*FABP1*), fibrinogen A (*FGA*), and transferrin (*TF*) as plots in the upper left region, whereas hESCs (ES01), hiPSCs (201B7), and fibroblasts exhibited negligible expression. Nevertheless, established clones AFB1-1, NGC1-1, and NGC1-2 also expressed ESC/iPSC-enriched genes including highly specific markers, *POU5F1*, *SOX2*, *NANOG*, *LIN28*, *SALL4*, and *TERT* as plots along the diagonal. Profiling plots also show that the gene expressions of the three clones were very similar to one another. The clones expressed hepatic genes much lower than adult hepatocytes did and expressed hESC/hiPSC-enriched genes at a level equivalent to hESCs (ES01, BG03, and H9) and hiPSCs (201B7) (see also S2 and S3 Figs). Cluster analysis using a heat map clearly shows that the established clones were categorized as an independent cluster and distinct from other pluripotent stem cells (ES01, BG03, H9, and 201B7), hepatic cells (adult hepatocytes and cell line HuH7), and fibroblasts (see also S4 Fig). Thus, the three established clones exhibited unique gene expression profiles different from those of typical hiPSCs and hESCs.

Such a new type of hiPSC, termed hiHSCs, is self-renewed and could be effectively expanded under coculture with the MEFs mainly in mTeSR1 medium and occasionally in ReproStem medium on gelatin-coated dishes. After collagenase treatment, hiHSCs were indistinguishable in morphology from typical hiPSCs under a conventional culture with the MEFs in ReproStem medium on gelatin-coated dishes. Otherwise, trypsinized hiHSCs formed many small colonies with clear edges when subcultured at a moderate cell density with the MEFs in mTeSR1 medium on gelatin-coated dishes. With the addition of Y-276322 to the media, hiHSCs were passaged with a recovery ratio of almost all. The colonies consisted of small cells and the surrounding flat cells under continuous subculture at a very high density (Fig 1D and S5 Fig). The population doubling time of the three clones was approximately 36–48 hr when the passages were split at 1:10 to 1:30 as single cells or very small clusters consisting of approximately 2 to 10 cells after trypsinization. Giemsa banding and multicolor FISH analyses confirm that each clone had a normal karyotype with no chromosomal translocation or deletion (S6 Fig). Clones AFB1-1 and NGC1-1 have been expanded over more than 85 and 70 passages, respectively, after the three-gene infection.

We further characterized hiHSCs by the detection of alkaline phosphatase (ALP) activity and fluorescent immunocytochemistry. Self-renewing hiHSCs were positive for ESC/hiPSC surface markers (SSEA-4, SSEA-3, TRA-1–60, and TRA-1–81) (S7 Fig), hESC/hiPSC-specific transcription factors, and hepatocyte-marker proteins (S1H and S1I Fig). Immune double-staining confirms the co-expression of both hESC/hiPSC-specific transcription factors (OCT3/4, SOX2, and NANOG) and hepatocyte-marker proteins (AFP, FABP1, ALB, CK8, and DLK1) (Fig 1E and S10 Fig). ALP activity stained the colonies with violet coloring (Fig 1E, lower right panel and S8 Fig, lowest right panel).

Self-renewing hiHSCs were the double positive cells of hESC/hiPSC and hepatocyte markers when subcultured at a moderate cell density. Nevertheless, single positive cells, which lost their ESC/iPSC markers (OCT3/4, NANOG, and SOX2), occasionally emerged around the double positive cells when the culture was refreshed with mTeSR1 medium every two days or every other day under continuous subculture at a very high density (Fig 1E and S9 Fig). Thus, hiHSCs were defined by their expression profiles of both hESC and hepatic markers.

### hiHSCs Markedly Enhance Expression of Hepatic Markers with Depletion of FGF-2

We investigated the differentiation potentials of hiHSCs by depletion of FGF-2 from ReproStem medium for an hESC/hiPSC culture without the addition of growth factors necessary for hepatic differentiation of hiPSCs. FGF-2 is an essential growth factor for the self-renewal of hESCs/hiPSCs [26]. In contrast, several growth factors, including FGF-2, have been needed as additional proteins for hepatic differentiation [27, 28]. In those studies, the growth-factor combinations needed for each complicated protocol were similar but not identical to one another. The resultant hepatocytes remain less robust in reproducibility, substantially incomparable, and partially immature. To determine the best combination of growth factors in each differentiation step, we needed to test the hepatic differentiation potentials of hiHSCs under a basal culture condition. Therefore, no exogenous differentiation growth factors were added to the ReproStem medium in any of the steps. To analyze hiHSCs from the different backgrounds of the starting cells, clone AFB1-1 from white female adult dermal fibroblasts and clone NGC1-1 from Japanese adult male gastric tissue-derived cells were employed for the differentiation culture.

**Fig 2:**
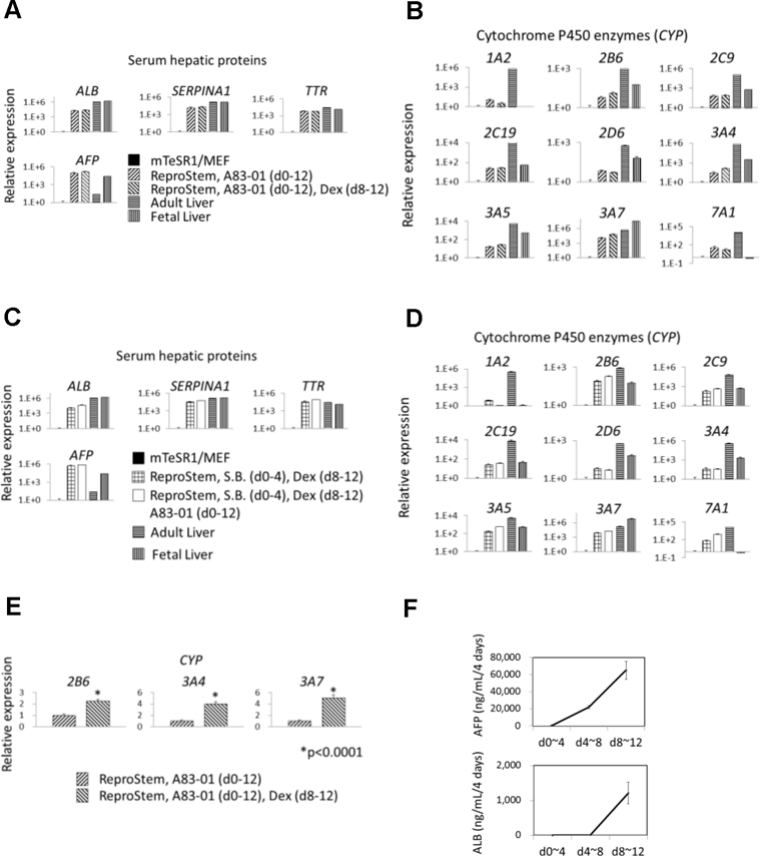
Hepatic differentiation of hiHSCs by depletion of FGF-2. Self-renewing hiHSCs, clones AFB1-1 and NGC1-1, differentiate into hepatocyte-like cells with the omission of FGF-2 from ReproStem medium. (**A–E**) Gene expression was analyzed by quantitative RT-PCR at day 12. Gene symbols are shown for serum hepatic proteins and cytochrome P450 enzymes. The terms of additions are indicated in parentheses. (**A–D**) Relative expression is shown with a logarithmic axis histogram. The expression is normalized to 1 in the self-renewing hiHSCs (mTeSR1/MEF) and compared to that of hepatocyte-like cells. Total RNAs of human fetal and adult livers were utilized as robust controls. Data are presented as mean+SEM and represent a minimum of three independent samples with at least two technical duplicates. (**A, B**) Clone AFB1-1 differentiated in the medium including an inhibitor (0.5 μM A83-01) of TGF-β signaling or the medium including 0.5 μM A83-01 plus 0.5 μM dexamethasone (Dex). See also S11 and S12 Figs. (**C, D**) Clone NGC1-1 differentiated in the medium including 0.5 μM sodium butyrate (S.B.) plus 0.5 μM Dex or the medium including 0.5 μM S.B. plus 0.5 μM Dex plus 0.5 μM A83-01. See also S13 and S14 Figs. (**E**) Clone AFB1-1 differentiated in the medium including 0.5 μM A83-01 or the medium including 0.5 μM A83-01 plus 0.5 μM Dex. Relative expression is normalized to 1 in the medium without Dex and is shown in the histogram. The expressions of cytochrome P450 enzymes (*CYP2B6*, *3A4*, and *3A7*) are induced by the addition of Dex. Asterisk indicates statistical significance as determined by *t* test. *p < 0.0001. (**F**) Release of human ALB and AFP was measured by ELISA on samples (supernatants of clone AFB1-1 differentiated with the medium including 0.5 μM A83-01) at three time points. Data are presented as mean±SEM and represent a minimum of three independent samples with at least two technical duplicates. See also Table 1.

Before the differentiation culture, hiHSCs were expanded under non-typical coculture with the MEFs in mTeSR1 medium on gelatin-coated dishes at a very high density. The cells were seeded with mTeSR1 medium on Matrigel-coated dishes, and they continued to culture in ReproStem medium without FGF-2 for 12 days. Interestingly, a high concentration of AFP was preliminarily detected in their culture supernatants. Therefore, we analyzed the gene expression in the cells by quantitative RT-PCR. As self-renewing hiHSCs expressed the genes of hepatic markers before the differentiation culture, the gene expressions were normalized to 1 in the self-renewing cells and compared to those of the differentiated cells at day 12. Beyond our expectations, clones AFB1-1 and NGC1-1 enhanced the gene expression of serum hepatic proteins (*ALB*, *SERPINA1*, *TTR*, and *AFP*) remarkably (Fig 2A and 2C, S11 Fig, and S13 Fig) and cytochrome P450 enzymes (*CYPs1A2*, *2B6*, *2C9*, *2C19*, *2D6*, *3A4*, *3A5*, *3A7*, and *7A1*) partially (Fig 2B and 2D, S12 Fig, and S14 Fig). In contrast to those hepatic genes, hESC/hiPSC-specific genes (*NANOG*, *OCT3/4*, and *SOX2*) reduced their expression of the differentiated cells compared with those of the self-renewing cells (S11 and S13 Figs), as expected. In a detailed comparison, the differentiated cells of clones AFB1-1 and NGC1-1 expressed *AFP* higher, *SERPINA1* and *TTR* similarly, and *ALB* lower in comparison with those of human adult and fetal livers (Fig 2A and 2C). The differentiated cells expressed *CYP3A7* similarly and other *CYPs* much lower compared to those of adult livers whereas those cells expressed *CYP7A1* much higher compared to those of fetal livers (Fig 2B and 2D). The addition of 0.5 μM dexamethasone into the ReproStem medium effectively promoted the hepatic specification and commitment of hiHSCs (Fig 2A and 2B, S11 Fig, and S12 Fig). Meanwhile, the addition caused statistically significant induction of the expression of *CYPs2B6*, *3A4*, and *3A7* (Fig 2E), suggesting a fetal hepatocyte-like phenotype. It was reported that a low dose of dexamethasone induces the expression of *CYP3A4* and *CYP3A7* in the fetal hepatocyte but not in the adult hepatocyte [29]. Otherwise, hiHSCs were differentiated under the culture with or without an inhibitor of TGF-β signaling, 0.5 μM A83-01. The inhibitor markedly enhanced the expression of hepatic genes except for *CYPs1A2*, *2D6*, and *3A4* (Fig 2C and 2D, S13 Fig, and S14 Fig) and it effectively reduced the expression of hESC/hiPSC-specific genes (S13 Fig). The result suggests that inhibition of TGF-β signaling in hiHSCs also promoted their hepatic specification and commitment.

**Table 1:** The secretion of AFP and ALB in vitro and in vivo.

Medium or animal	In vitro or in vivo	Cell number	Day	AFP (ng/mL)	ALB (ng/mL)	AFP/ALB
ReproStem + A83-01	In vitro	1.0E+6	0~4	529	Not detected	∞
ReproStem + A83-01	In vitro	1.0E+6	4~8	22,100	7	3,157.1
ReproStem + A83-01	In vitro	1.0E+6	8~12	65,000	1,210	53.7
ReproStem + A83-01	In vitro	0	-	Not detected	Not detected	-
Essential 6	In vitro	1.0E+6	0~4	140	9	15.6
Essential 6	In vitro	1.0E+6	4~8	19,900	30	663.3
Essential 6	In vitro	1.0E+6	8~12	65,600	450	145.8
Essential 6	In vitro	0	-	Not detected	Not detected	-
NOD/SCID mouse	In vivo	1.0E+7	0~66	2,879	1,483	1.9
NOD/SCID mouse	In vivo	2.0E+7	0~83	1,747	615	2.8
NOD/SCID mouse	In vivo	3.7E+7	0~84	2,397	1,593	1.5
NOD/SCID mouse	In vivo	0	-	Not detected	Not detected	-

To confirm the hepatic differentiation of hiHSCs, we examined the protein production of human AFP and ALB from the differentiating cells. AFP and ALB were measured by enzyme-linked immunosorbent assays (ELISAs) on the supernatants of hiHSCs differentiating with ReproStem medium including 0.5 μM A83-01. The differentiating cells increased the secretion of AFP at a very high concentration and markedly secreted ALB between days 8 and 12 after the omission of FGF-2 (Fig 2F and Table 1). Thus, hiHSCs could robustly differentiate into hepatocyte-like cells even under several variations of culture simply by omitting the addition of FGF-2 to the medium. Unlike a typical hiPSC culture, the hepatic specification of hiHSCs was not due to the backgrounds of starting cells.

### hiHSCs Autonomously Differentiate into Hepatocyte-Like Cells without Any Exogenous Factors

To confirm reproducibly whether hiHSCs differentiate into hepatocyte-like cells under the culture without any unknown components or chemical compounds, we adopted an Essential 6 (E6) medium that consists simply of insulin, transferrin, selenium, and L-ascorbic acid in DMEM/F12 medium. This is a medium in which TGF-β1 and FGF-2 have been eliminated from the Essential 8 medium defined for an hESC/hiPSC culture [30]. To intensively analyze hiHSCs from the accessible tissue of the starting cells for medical applications, we focused on clone AFB1-1 from dermal fibroblasts rather than clone NGC1-1 from gastric tissue-derived cells. Likewise, hiHSCs were seeded with mTeSR1 medium on Matrigel-coated dishes, and the seeded cells continued to culture in Essential 6 medium.

**Fig 3:**
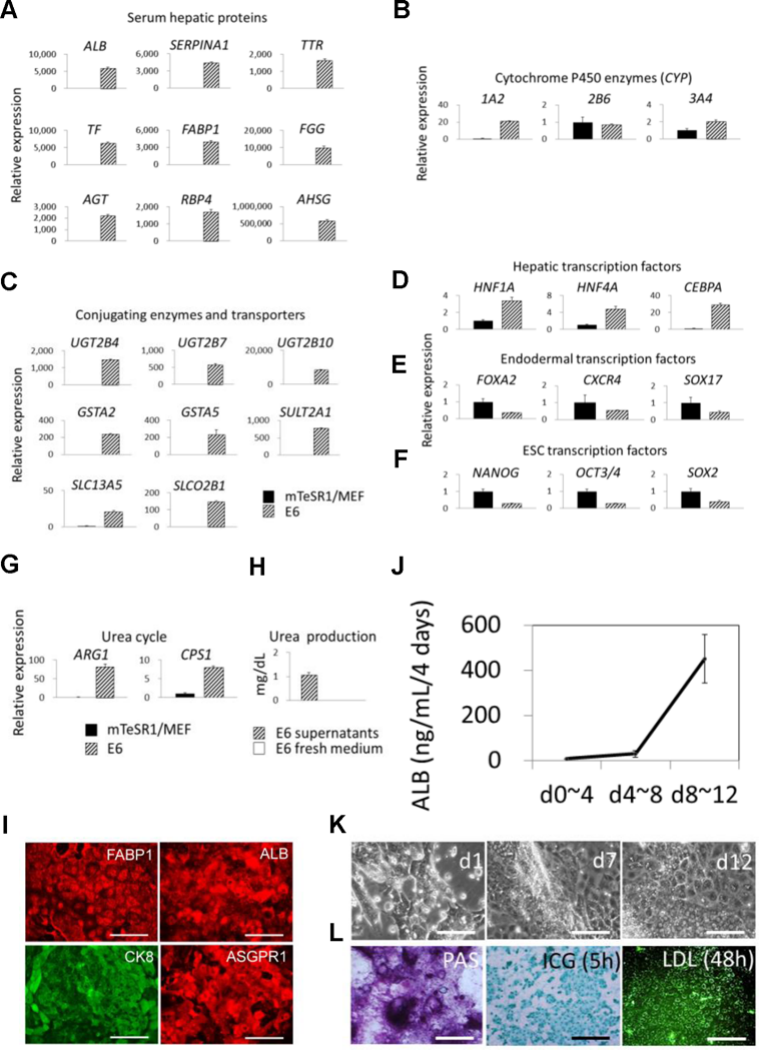
Autonomous hepatic differentiation of hiHSCs in Essential 6 medium. Self-renewing hiHSCs (clone AFB1-1) differentiate into hepatocyte-like cells in defined Essential 6 medium that consists simply of insulin, transferrin, selenium, and L-ascorbic acid in DMEM/F12 medium. (**A–G**) Gene expression was analyzed by quantitative RT-PCR at day 12. Relative expression is shown in the histogram. The expression is normalized to 1 in the self-renewing hiHSCs (mTeSR1/MEF) and compared to that of hepatocyte-like cells (E6). Gene symbols are shown for (**A**) serum hepatic proteins, (**B**) cytochrome P450 enzymes, (**C**) conjugating enzymes and transporters, (**D**) hepatic transcription factors, (**E**) endodermal transcription factors, (**F**) ESC transcription factors, and (**G**) urea cycle-related enzymes. Data are presented as mean+SEM and represent a minimum of three independent samples with at least two technical duplicates. (**H**) Urea production was measured by urea nitrogen detection kits on the supernatants of cells at day 12. E6 fresh medium was also measured as a negative control. Data are presented as mean+SEM and represent a minimum of three independent samples. (**I**) Immunofluorescent staining of hepatocyte-like cells for FABP1, ALB, CK8, and ASGPR1. Scale bar represents 100 μm. (**J**) Release of ALB was measured by ELISA at three time points. Data are presented as mean±SEM and represent a minimum of three independent samples with at least two technical duplicates. See also Table 1. (**K**) Phase contrast micrographs showing the morphology of differentiating cells. Scale bar represents 100 μm. (**L**) At day 12, hepatocyte-like cells are shown to store glycogen by Periodic Acid—Schiff (PAS) staining. Otherwise, the cells were stained to uptake indocyanine green (ICG) for 5 hr and BODIPY FL–labeled low density lipoprotein (LDL) for 48 hr. Scale bar represents 100 μm.

We analyzed the gene expression in the cells by quantitative RT-PCR. Even cultured in simplified E6 medium for 12 days, the differentiated cells markedly enhanced the gene expression of serum hepatic proteins (*ALB*, *SERPINA1*, *TTR*, *TF*, *FABP1*, *FGG*, *AGT*, *RBP4*, and *AHSG*) and conjugating enzymes and transporters (*UGT2B4*, *UGT2B7*, *UGT2B10*, *GSTA2*, *GSTA5*, *SULT2A1*, *SLC13A5*, and *SLCO2B1*) (Fig 3A and 3C). The gene expression of the urea cycle-related enzymes (*ARG1* and *CPS1*) of the differentiated cells was also enhanced (Fig 3G), and their urea productions into supernatants were confirmed by urea nitrogen detection (Fig 3H). Nevertheless, the gene expression of *CYPs2B6* and *3A4* was not effectively enhanced in the E6 medium (Fig 3B). We further analyzed the gene expression of hepatic transcription factors (*HNF1A*, *HNF4A*, and *CEBPA*), endodermal transcription factors (*FOXA2*, *CXCR4*, and *SOX17*), and hESC/hiPSC-specific transcription factors (*NANOG*, *OCT3/4*, and *SOX2*) in comparison with the self-renewing cells and the differentiated cells. The resultant hepatocyte-like cells enhanced the gene expression of *HNF1A*, *HNF4A*, and *CEBPA* (Fig 3D), whereas the gene expression of *FOXA2*, *CXCR4*, *SOX17*, *NANOG*, *OCT3/4*, and *SOX2* was reduced after differentiation (Fig 3E and 3F).

Furthermore, immunofluorescence staining shows that the hepatocyte-like cells are strongly positive for the typical hepatocyte-specific marker proteins FABP1, ALB, CK8, and asialoglycoprotein receptor 1 (ASGPR1) (Fig 3I). ELISAs confirm that the differentiating cells markedly secreted ALB even in supernatants of the E6 medium between days 8 and 12 (Fig 3J and Table 1). Phase contrast micrographs show that the differentiating cells mostly became flat cells with a moderate nucleus-to-cytoplasm ratio through the culture (Fig 3K). The hepatocyte-like cells could store glycogen and uptake indocyanine green and low-density lipoprotein (Fig 3L). Altogether, these results conclusively suggest that hiHSCs autonomously differentiated into hepatocyte-like cells under the culture without any growth factors or chemical compounds necessary for hepatic specification and commitment.

### hiHSCs Preferentially Differentiate toward a Hepatic Lineage

**Fig 4:**
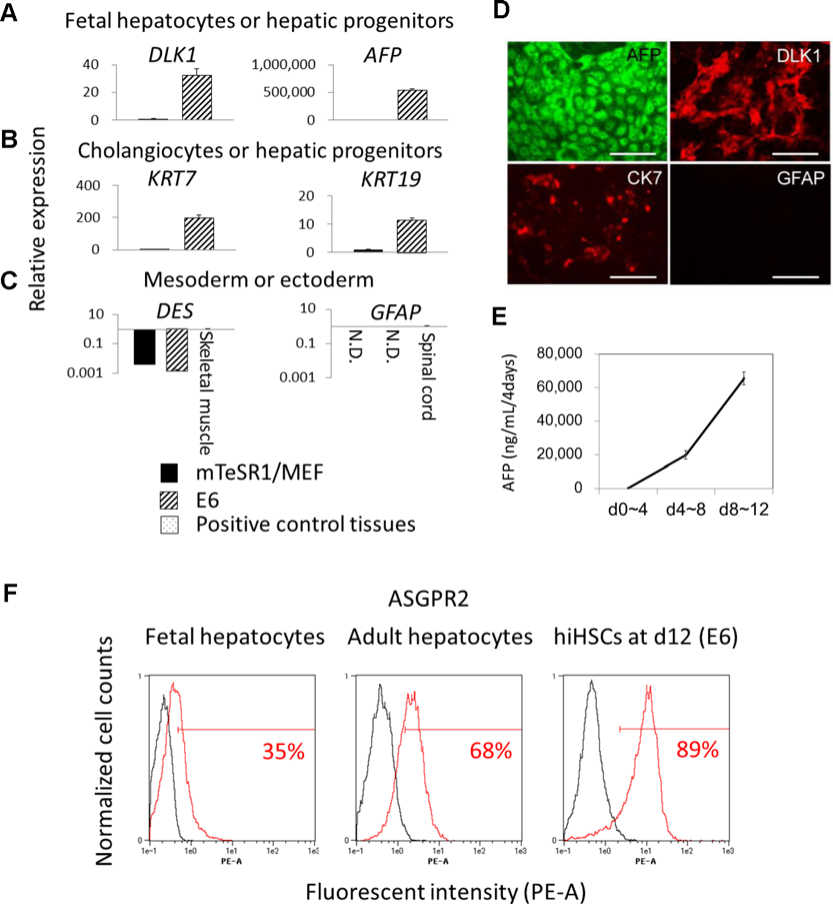
Preferential hepatic specification of hiHSCs in Essential 6 medium. Self-renewing hiHSCs (clone AFB1-1) preferentially orient toward hepatic specification in Essential 6 medium without any exogenous differentiation factors. See also Fig 3. (**A–C**) Gene expression was analyzed by quantitative RT-PCR. (**A**) *DLK1* and *AFP*, (**B**) *KRT7* and *KRT19*, and (**C**) *DES* and *GFAP* are shown as gene symbols. Total RNAs of human skeletal muscles and spinal cords were utilized as positive controls for the expression of *DES* and *GFAP*, respectively. Data are presented as mean+SEM and represent a minimum of three independent samples with at least two technical duplicates. (**D**) Immunofluorescent staining for AFP, DLK1, CK7, and GFAP. Scale bar represents 100 μm. (**E**) Release of AFP was measured by ELISA at three time points. Data are presented as mean±SEM and represent a minimum of three independent samples with at least two technical duplicates. See also Table 1. (**F**) Flow cytometry analyses of ASGPR2-positive cells. Data are presented as the mean and represent a minimum of three independent samples.

To investigate the hepatic maturation and directivity of hiHSCs in E6 medium, we analyzed the gene expression of immature hepatic and non-hepatic markers. The differentiated cells enhanced the gene expression of hepatoblast marker *DLK1*, cholangiocyte and hepatic progenitor markers *KRT7* and *KRT19* [31, 32, 18], and especially fetal hepatocyte marker *AFP* to approximately 500,000-fold (Fig 4A and 4B). The results suggest that the differentiated cells were partially immature hepatocyte-like cells, such as fetal hepatocyte-like cells. We further analyzed the gene expression of mesodermal (desmin/*DES*) and ectodermal (glial fibrillary acid protein/*GFAP*) markers to investigate whether hiHSCs differentiated toward other lineages (Fig 4C). In contrast to hepatic markers, gene expression of *DES* was reduced after differentiation, and the relative expression was much lower than that of skeletal muscle. Likewise, RT-PCR analysis could not detect gene expression of *GFAP*. Immunofluorescence staining confirms that the differentiated cells were immature hepatocytes, almost all positive for AFP and DLK1 and partially positive for cytokeratin 7 (CK7), whereas they were negative for GFAP (Fig 4D). ELISAs further verify that the differentiating cells markedly secreted AFP (Fig 4E and Table 1), which is a fetal and immature hepatocyte marker protein.

To investigate at a single cell level whether hiHSCs differentiated toward only a hepatic lineage, we adopted flow cytometry analyses for the cell surface antigen of the asialoglycoprotein receptor (ASGPR) that is specifically expressed in hepatocytes to a significant degree [33, 34]. Flow cytometry analyses reveal that the differentiated cells were approximately 90% positive for ASGPR2, whereas fetal hepatocytes and adult hepatocytes were 35% and 68% positive for an antigen of ASGPR2, respectively (Fig 4F). Collectively, hiHSCs preferentially differentiated into partially immature hepatocyte-like cells even in Essential 6 defined medium without any exogenous factors.

### Transplantation of hiHSCs Causes Hepatic Function in Mice

**Fig 5:**
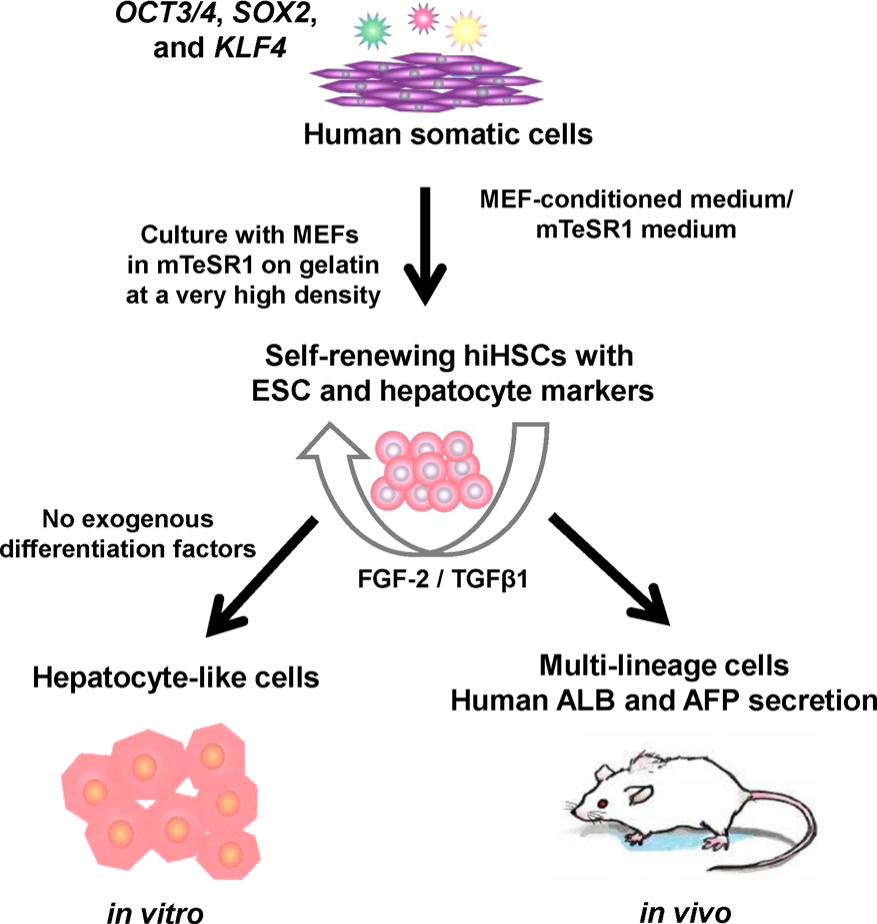
Generation, self-renewal, and differentiation of hiHSCs. Generation, self-renewal, and differentiation of hiHSCs were schematically illustrated. hiHSCs were generated and expanded as a new type of hiPSC from human somatic cells by gene transfer of defined factors and with some modifications of their culture conditions. The autonomous hepatic specification of hiHSCs was due to their culture conditions (coculture with MEF in mTeSR1 medium at a very high density) in self-renewal rather than in differentiation. Self-renewing hiHSCs expressed markers of both hESCs and hepatocytes and then autonomously differentiated to hepatocyte-like cells in a defined minimum medium without FGF-2 and TGF-β1in vitro. Otherwise, in vivo differentiated hiHSCs gave rise not only to hepatocyte-like cells but also to other multi-lineage cells and then secreted human ALB in mice.

To confirm the differentiation potentials of hiHSCs in vivo, 10 to 37 million of the self-renewing hiHSCs were mixed with Matrigel, and the mixture was injected subcutaneously into NOD/SCID mice. Blood was drawn, and the resultant teratomas were isolated when sacrificed at 66, 83, and 84 days post-transplantation. We adopted a method to specify and quantify human hepatic markers as a whole in mice. Since most highly specific hepatic proteins are serum proteins that secrete into the blood circulation of mice, we performed ELISAs to specifically measure the mouse serum levels of human ALB and AFP. Human ALB and AFP were detected between the concentrations of approximately 0.6–1.6 and 1.7–2.9 μg/mL, respectively, in the sera of mice bearing teratomas but not in those of normal mice (Table 1). Thus, the secretion of human ALB as a mature hepatic function remained at a concentration of approximately 1.6 μg/mL in the blood circulation even at 84 days after transplantation. The immunohistochemical analyses validate that the transplantation of hiHSCs caused teratomas containing tissues representative of the three germ layers, including gut epithelium-like structures, cartilage-like structures, and neural epithelium-like structures (S15 Fig). Altogether, these results suggest that in vivo differentiated hiHSCs gave rise not only to hepatocyte-like cells but also to other multi-lineage cells and caused hepatic function in mice (Fig 5).

## Discussion

Here, we describe self-renewing hiHSCs that autonomously differentiate into hepatocyte-like cells without any exogenous factors (Fig 5). The molecular mechanism underlying the autonomous hepatic specification of hiHSCs was partially elucidated. It was conceivable that the mechanism underlying the preference for such a specification was as follows. The hiHSCs expressed not only the genes of the core pluripotency transcription factors (*NANOG*, *OCT3/4*, and *SOX2*) but also the genes of hepatic markers under a self-renewing culture. Under a culture with the omission of FGF-2 and TGF-β1, the gene expression of the core pluripotency transcription factors and endodermal transcription factors was reduced, whereas that of hepatic transcription factors and hepatic markers was enhanced in differentiating cells. Therefore, it is likely that the specification of hiHSCs was directed by hepatic transcription factors, the gene products of *HNF1A*, *HNF4A*, and *CEBPA*. On the other hand, it seems that the hepatic preference of self-renewing hiHSCs was destined by their expression profiles of hepatic markers. Meanwhile, the gene products of *NANOG*, *OCT3/4*, and *SOX2* might play a role in hiHSCs as transcription factors for self-renewal rather than pluripotency or undifferentiation.

Most importantly, our study suggests that the robust reproducibility for the hepatic specification of hiHSCs is due to their culture conditions (coculture with MEFs in mTeSR1 medium at a very high density) in self-renewal rather than in differentiation. Nevertheless, our culture is only a little variation from several protocols that have been described previously in the generation of hiPSCs [35]. In hepatic differentiation for only 12 days, it is essential that hiHSCs have routinely been confluent at a very high density when they have been passaged at day 7 of the self-renewal culture. Continuous passaging of self-renewing hiHSCs at moderate confluency results in extremely highly proliferating cells that need a differentiation culture for three or more weeks. In other words, AFP is produced less in the case that hiHSCs are poorly differentiated when a continuous self-renewal culture of them is passaged at moderate confluency. The extent of AFP production is correlated to the expression of hepatic genes, including *AFP*, *ALB*, *TTR*, and *SERPINA1*. The measurement of AFP is useful as an indicator of the hepatic specification of hiHSCs during differentiation culture, as the secretion of this fetal hepatic protein precedes that of ALB in their culture supernatants, and it can be detected even four days after the culture. In addition, a high level of AFP production strongly suggests that hiHSC-differentiated cells were fetal hepatocyte-like cells. Meanwhile, CYP activities of the hepatocyte-like cells were not always reproducibly active under culture in media employed for autonomous hepatic differentiation without exogenous differentiation factors. Therefore, we need to define a medium for the maturation of hiHSCs into fully differentiated functional hepatocytes with CYP activities. In our future study, it would be of great significance to extensively compare hiHSC- differentiated cells with non-cultured or cultured authentic hepatocytes and livers based on various criteria, including genome-wide gene expression, proteome [36], and CYP activities.

Nevertheless, there were experimental issues relating to the identity of cells, as hiHSC- differentiated cells were hepatocyte-like cells rather than fetal or adult liver-derived hepatocytes. Even adult hepatocytes prepared from different donors have phenotypic variations from one another, and fetal hepatocytes have huge variations in different developmental stages. In addition, hepatocytes undergo extreme changes to their phenotypes including hepatic gene expression under different culture conditions. Moreover, the positivity of the ASGPR2 protein expressing on the cell surface would be affected by enzymatically preparing single cells for flow cytometry analysis, implying that the negative percentage for ASGPR2 did not always indicate other lineage cells such as CK7-positive cholangiocytes. Further, it is well known that many hepatocyte lots prepared from livers are non-adhesive cells, and adhesive hepatocytes vary widely in their plating efficiency. Therefore, we adopted adult and fetal livers as robust controls of relative gene expression, although the expression profiles of hiHSC-differentiated hepatocyte-like cells were not identical to or similar to those of the livers as a whole, although livers are substantially different from cultured hepatocytes. Otherwise, our study focuses on the assertion that hepatic specification of hiHSCs is not due to exogenous differentiation factors. Therefore, we employed a defined minimum medium and ESC medium (without FGF-2) to demonstrate hepatic specification (immature differentiation) without exogenous factors such as growth factors and serum. Obviously, such media would not be applicable to a culture for adult or fetal liver-derived hepatocytes; therefore, it would not be of significance in directly comparing the secretion of ALB and AFP between our hepatocyte-like cells and liver-derived hepatocytes under such a culture. In addition, it could be inferred that the secretion of ALB by adult hepatocytes in an appropriate medium varies widely with their different lots and culture periods [37].

Donor differences in hiPSC generation are considered to be an important determinant of the propensity for differentiation and the transcriptional variation between their lines [12, 38]. Genetic and epigenetic variations in hiPSCs have also been reported [39, 40]. In addition, it was shown that long-term culture of mouse iPSCs or hiPSCs reduces inherited histone or DNA methylation states [41, 42]. This suggests that iPSCs lose the epigenetic background inherited from the starting cells and resemble ESCs through a typical self-renewal culture. It is conceivable that some modification of a self-renewal culture conversely determines the fate of hiPSC differentiation toward a specific lineage. Indeed, the self-renewal of hiHSCs under non-typical coculture is more important for robust hepatic differentiation than their differentiation culture. In addition, the culture condition of hiHSC generation is as essential as that of their self-renewal to achieve hepatic specification. Evidently, typical hiPSCs in generation have not yet given rise to hiHSCs under our self-renewal culture to exhibit the autonomous hepatic differentiation. In other words, typical hiPSCs did not substantially expand under such a culture, and those cells resulted in cell death under our differentiation culture. Unexpectedly, there are no obvious donor differences between Japanese male gastric and white female dermal tissues in the autonomous hepatic differentiation of hiHSCs. Therefore, it seems that the hepatic directivity of hiHSCs is not attributable to the donors or tissues of the starting cells. Although the DNA methylation characteristic of hepatic propensity has not yet been elucidated [12], our data show that the autonomous hepatic specification of self-renewing hiHSCs is directly defined by their expression profiles of hepatic markers. Generally, it is likely that culture conditions in both the generation and self-renewal of hiPSCs play pivotal roles in determining their directivity to a specific lineage.

The transplantation of self-renewing hiHSCs markedly caused the secretion of human ALB into the blood circulation of mice through in vivo differentiation. The resultant concentrations of human ALB were greater than those of human liver-derived organoid cells [43] or comparable to those of hiPSC-derived liver buds [10], although the transplantations were performed in each distinct experimental setting. Interestingly, the ratio of the secretion of AFP versus ALB decreased more in vivo than in vitro (Table 1). The ratio might be an indicator of hepatic maturation, as AFP and ALB are fetal and adult hepatic serum proteins, respectively. Therefore, the engraftment of in vivo differentiated hepatocyte-like cells might be advantageous for hepatic maturation. We designed the transplantation of hiHSCs themselves as our in vivo study because it has been reported that the engraftment and human ALB secretion as a hepatic function of hiPSC-derived hepatocyte-like cells had little effect on the liver injury of mice [14, 18, 44, 45, 46]. Consequently, it is likely that the engraftment of in vivo differentiated hepatocyte-like cells coexisting with other multi-lineage cells profitably secreted human ALB in mice, as did the engraftment of hiPSC-derived liver buds [10]. It seems that many kinds of murine growth factors stimulated hiHSCs that gave rise not only to hepatocyte-like cells but also to other multi-lineage cells in mice. The resultant multi-lineage cells might facilitate the maturation of hepatocyte-like cells. Nevertheless, it would be essential for the orthotopic engraftment to identify unknown essential factors or define a specific culture condition to differentiate hiHSCs into fully functional hepatocytes rather than hepatocyte-like cells. To validate hiHSC-derived fully functional hepatocytes, we need to very efficiently engraft those cells into liver injury models of super-immunodeficient mice, such as FRG mice [47] and TK-MOG mice [48], in our future study.

## Conclusions

Highly expandable hiHSCs autonomously differentiate into hepatocyte-like cells without any additional growth factors, chemical compounds, or gene transfer. Thus, hiHSCs exhibit their remarkable hepatic directivity in vitro. This suggests the feasibility of preparing large quantities of hepatocytes as a convenient and inexpensive hiPSC differentiation. Our study also suggests the necessity of optimizing culture conditions to generate other specific lineage-oriented hiPSCs, allowing for a very simple differentiation.

## Supporting Information

S1 Fig Heat map of the expression profiles of the genes defined by the ISCI. The established clones were analyzed by microarray to compare with fibroblasts, hESCs (hES-ES01, hES-BG03, and hES-H9), hiPSCs (hiPS-201B7), a hepatocellular carcinoma cell line (HuH-7), and a human adult hepatocyte. Normalized fluorescent intensity values range from red (high) to blue (low) coloring, and the resultant heat map is shown with gene symbols. (TIF) The gene list, which was defined by the International Stem Cell Initiative (ISCI), is shown in S4 Table.
(TIFF)

S2 Fig Heat map of the expression profiles of hepatic genes. The established clones were analyzed by microarray to compare with fibroblasts, hESCs (hES-ES01, hES-BG03, and hES-H9), hiPSCs (hiPS-201B7), a hepatocellular carcinoma cell line (HuH-7), and a human adult hepatocyte. Normalized fluorescent intensity values range from red (high) to blue (low) coloring, and the resultant heat map is shown with gene symbols. (TIF) The list of hepatic genes is shown in S5 Table.
(TIF)

S3 Fig Heat map of the expression profiles of hESC-enriched genes. The established clones were analyzed by microarray to compare with fibroblasts, hESCs (hES-ES01, hES-BG03, and hES-H9), hiPSCs (hiPS-201B7), a hepatocellular carcinoma cell line (HuH-7), and a human adult hepatocyte. Normalized fluorescent intensity values range from red (high) to blue (low) coloring, and the resultant heat map is shown with gene symbols. (TIF) The list of hESC-enriched genes is shown in S6 Table.
(TIF)

S4 Fig Heat map of the expression profiles of both hESC-enriched genes and hepatic genes. The established clones were analyzed by microarray to compare with fibroblasts, hESCs (hES-ES01, hES-BG03, and hES-H9), hiPSCs (hiPS-201B7), a hepatocellular carcinoma cell line (HuH-7), and a human adult hepatocyte. Normalized fluorescent intensity values range from red (high) to blue (low) coloring, and the resultant heat map is shown with gene symbols. (TIF) The combined list of both hESC-enriched genes and hepatic genes is shown in S7 Table.
(TIF)

S5 Fig Phase contrast micrographs showing the morphology of clone NGC1-1. Phase contrast micrographs show the morphology of clone NGC1-1 at days 1, 4, and 6 after passage. Scale bar represents 100 μm. (TIF)
(TIF)

S6 Fig Giemsa banding and multicolor FISH of each hiHSC clone. Representative image of each clone is shown as follows: (Upper panels) NGC1-1 (46XY), (middle panels) NGC1-2 (46XY), and (lower panels) AFB1-1 (46XX). Fifty cells per clone were evaluated (Giemsa banding). Ten cells per clone were evaluated (multicolor FISH analysis). (TIF)
(TIF)

S7 Fig Immunostaining of clone AFB1-1 with hESC markers. Cells were stained with SSEA-4, TRA-1-60, SSEA-3, and TRA-1-81. Nuclei were stained with Hoechst 33452. Scale bar represents 50 μm. (TIF)
(TIF)

S8 Fig Staining of clone AFB1-1 with hepatocyte or hESC markers. Cells were stained with ALB, AFP, CK8, DLK1, FABP1, SOX2, NANOG, and alkaline phosphatase (ALP). Nuclei were stained with Hoechst 33452. Scale bar represents 50 μm. (TIF)
(TIF)

S9 Fig Double-staining of clone AFB1-1 with hepatocyte and hESC markers. Immunostaining confirms the co-expression of NANOG and DLK1, CK8 and NANOG, or CK8 and SOX2. Scale bar represents 100 μm. (TIF)
(TIF)

S10 Fig Double-staining of clone AFB1-1 with SOX2 and ALB or NANOG and FABP1. Immunostaining confirms the co-expression of SOX2 and ALB or NANOG and FABP1. Scale bar represents 50 μm. (TIF)
(TIF)

S11 Fig Gene expression of serum hepatic proteins and hESC-specific transcription factors. Clone AFB1-1 was cultured in the medium including 0.5 μM A-83-01 or the medium including 0.5 μM A-83-01 plus 0.5 μM dexamethasone (Dex) with the omission of FGF-2 from ReproStem medium. Gene expression was analyzed by quantitative RT-PCR at day 12 of the differentiation culture on samples. The expression was normalized to 1 in the self-renewing hiHSCs (mTeSR1/MEF) and compared to differentiated cells. Relative expression is shown as the histogram with the linear scale. The terms of additions are indicated in parentheses. Data are presented as mean+SEM and represent a minimum of three independent samples with at least two technical duplicates. (TIF) See also Fig 2A.
(TIF)

S12 Fig Gene expression of cytochrome P450 enzymes. Clone AFB1-1 was cultured in the medium including 0.5 μM A-83-01 or the medium including 0.5 μM A-83-01 plus 0.5 μM dexamethasone (Dex) with the omission of FGF-2 from ReproStem medium. Gene expression was analyzed by quantitative RT-PCR at day 12 of the differentiation culture on samples. The expression was normalized to 1 in the self-renewing hiHSCs (mTeSR1/MEF) and compared to differentiated cells. Relative expression is shown as the histogram with the linear scale. The terms of additions are indicated in parentheses. Data are presented as mean+SEM and represent a minimum of three independent samples with at least two technical duplicates. (TIF) See also Fig 2B.
(TIF)

S13 Fig Gene expression of serum hepatic proteins and hESC-specific transcription factors. Clone NGC1-1 was cultured in the medium including 0.5 μM S.B. plus 0.5 μM Dex or the medium including 0.5 μM S.B. plus 0.5 μM Dex plus 0.5 μM A-83-01 with the omission of FGF-2 from ReproStem medium. Gene expression was analyzed by quantitative RT-PCR at day 12 of the differentiation culture on samples. The expression was normalized to 1 in the self-renewing hiHSCs (mTeSR1/MEF) and compared to differentiated cells. Relative expression is shown as the histogram with the linear scale. The terms of additions are indicated in parentheses. Data are presented as mean+SEM and represent a minimum of three independent samples with at least two technical duplicates. (TIF) See also Fig 2C.
(TIF)

S14 Fig Gene expression of cytochrome P450 enzymes. Clone NGC1-1 was cultured in the medium including 0.5 μM S.B. plus 0.5 μM Dex or the medium including 0.5 μM S.B. plus 0.5 μM Dex plus 0.5 μM A-83-01 with the omission of FGF-2 from ReproStem medium. Gene expression was analyzed by quantitative RT-PCR at day 12 of the differentiation culture on samples. The expression was normalized to 1 in the self-renewing hiHSCs (mTeSR1/MEF) and compared to differentiated cells. Relative expression is shown as the histogram with the linear scale. The terms of additions are indicated in parentheses. Data are presented as mean+SEM and represent a minimum of three independent samples with at least two technical duplicates. (TIF) See also Fig 2D.
(TIF)

S15 Fig Staining of tissue sections from a teratoma with hematoxylin and eosin. Ten million hiHSCs (clone AFB1-1) mixed with Matrigel were injected subcutaneously into an NOD/SCID mouse. The resultant teratoma was isolated when sacrificed at 66 days post-transplantation. Tissue sections from the teratoma were stained with hematoxylin and eosin. The teratoma contained tissues representative of the three germ layers at lower magnification (upper left panel). Shown are gut epithelium-like structures (upper right panel), cartilage-like structures (lower left panel), and neural epithelium-like structures (lower right panel) at a higher magnification. Scale bar represents 100 μm. (TIF)
(TIF)

S1 Table Genotyping of alleles at HLA loci. (XLSX)
(XLSX)

S2 Table HLA types equivalent to genotyping. (XLSX)
(XLSX)

S3 Table STR genotyping. (XLSX)
(XLSX)

S4 Table List of genes defined by the International Stem Cell Initiative. (XLSX)
(XLSX)

S5 Table List of hepatic genes. (XLSX)
(XLSX)

S6 Table List of hESC-enriched genes. (XLSX)
(XLSX)

S7 Table Combined list of hepatic genes and hESC-enriched genes. (XLSX)
(XLSX)

S8 Table Real-time PCR primer list. (XLSX)
(XLSX)

S9 Table Primary antibody list. (XLSX)
(XLSX)

S10 Table Secondary antibody list. (XLSX)
(XLSX)
