# Supplementary figures and images for: Human Induced Hepatic Lineage-Oriented Stem Cells: Autonomous Specification of Human iPS Cells toward Hepatocyte-Like Cells without Any Exogenous Differentiation Factors
Source: PLoS One. 2015 Apr 13;10(4):e0123193. doi: 10.1371/journal.pone.0123193 (PMC4395359; doi:10.1371/journal.pone.0123193)

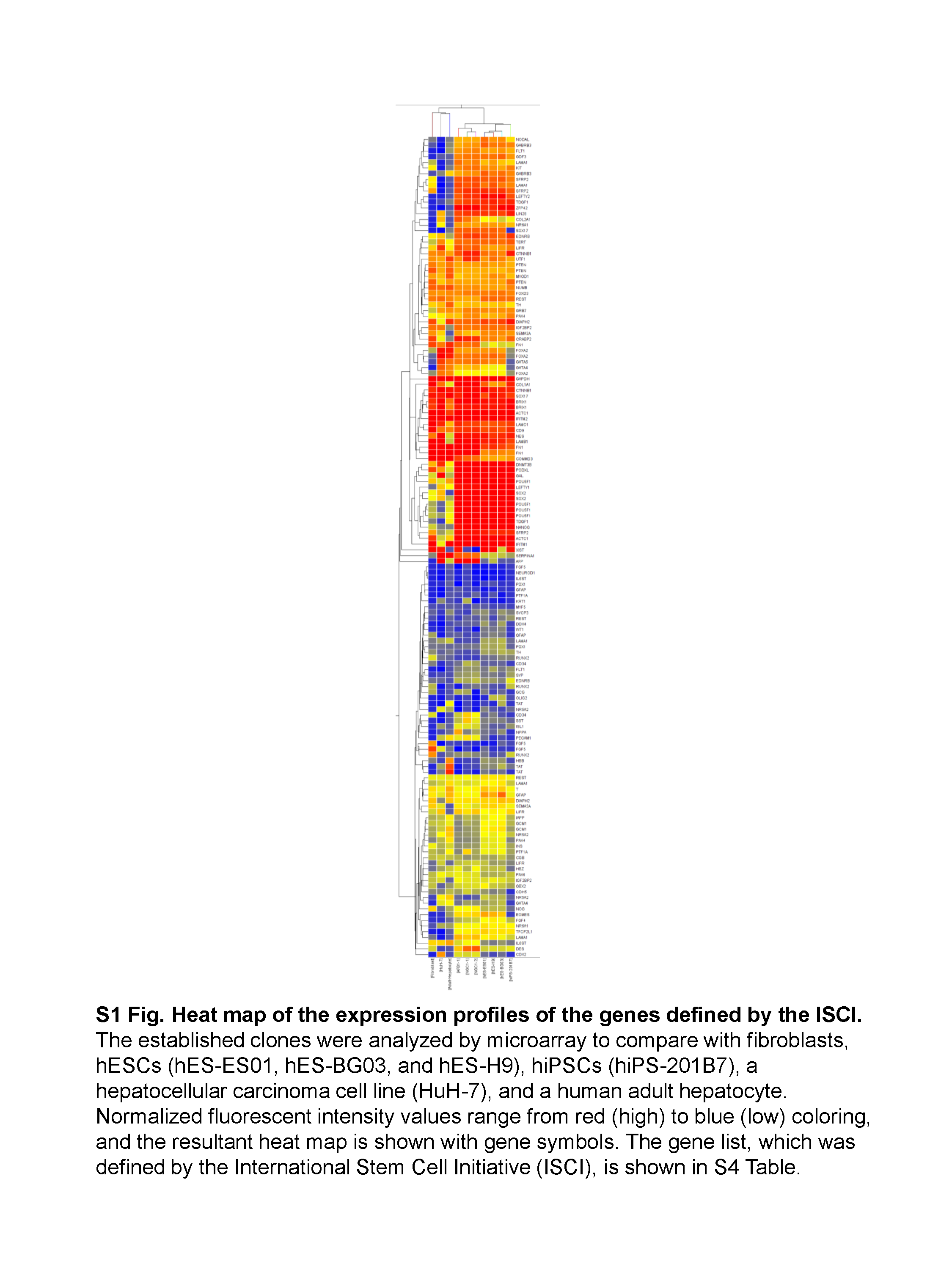

Supplement: S1 Fig — The established clones were analyzed by microarray to compare with fibroblasts, hESCs (hES-ES01, hES-BG03, and hES-H9), hiPSCs (hiPS-201B7), a hepatocellular carcinoma cell line (HuH-7), and a human adult hepatocyte. Normalized fluorescent intensity values range from red (high) to blue (low) coloring, and the resultant heat map is shown with gene symbols. (TIF) The gene list, which was defined by the International Stem Cell Initiative (ISCI), is shown in S4 Table. (TIFF) [file pone.0123193.s001.tiff]

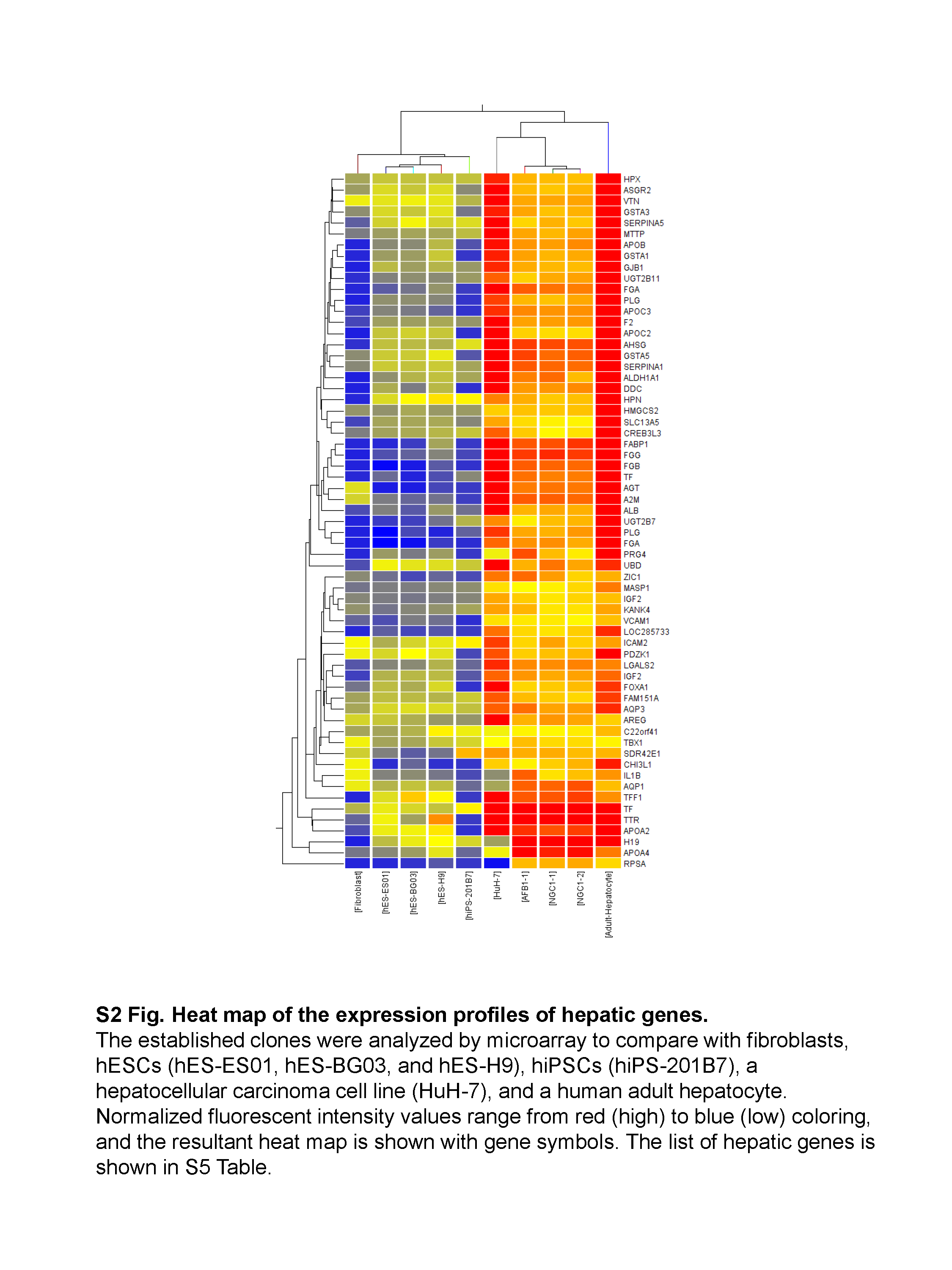

Supplement: S2 Fig — The established clones were analyzed by microarray to compare with fibroblasts, hESCs (hES-ES01, hES-BG03, and hES-H9), hiPSCs (hiPS-201B7), a hepatocellular carcinoma cell line (HuH-7), and a human adult hepatocyte. Normalized fluorescent intensity values range from red (high) to blue (low) coloring, and the resultant heat map is shown with gene symbols. (TIF) The list of hepatic genes is shown in S5 Table. (TIF) [file pone.0123193.s002.tif]

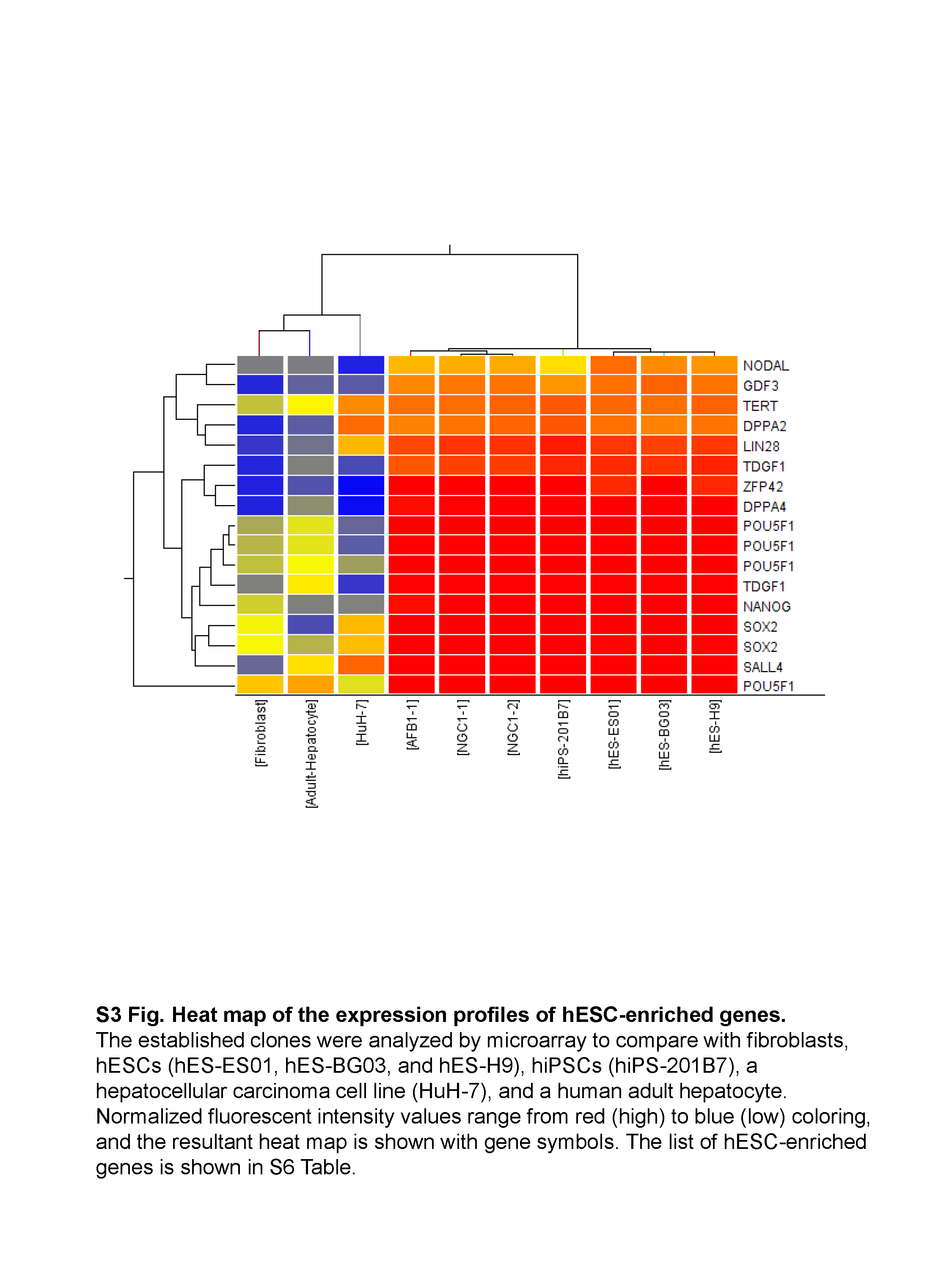

Supplement: S3 Fig — The established clones were analyzed by microarray to compare with fibroblasts, hESCs (hES-ES01, hES-BG03, and hES-H9), hiPSCs (hiPS-201B7), a hepatocellular carcinoma cell line (HuH-7), and a human adult hepatocyte. Normalized fluorescent intensity values range from red (high) to blue (low) coloring, and the resultant heat map is shown with gene symbols. (TIF) The list of hESC-enriched genes is shown in S6 Table. (TIF) [file pone.0123193.s003.tif]

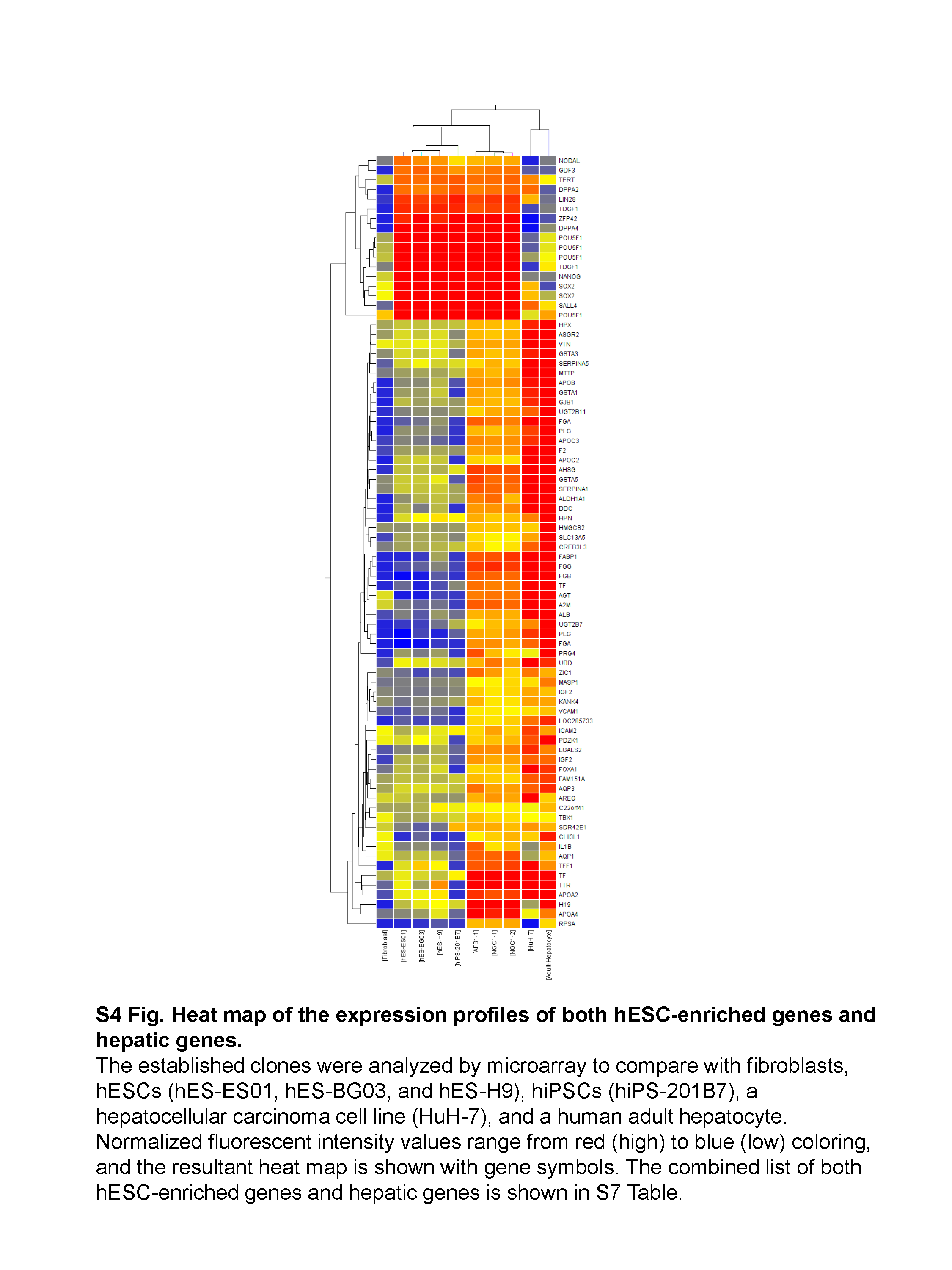

Supplement: S4 Fig — The established clones were analyzed by microarray to compare with fibroblasts, hESCs (hES-ES01, hES-BG03, and hES-H9), hiPSCs (hiPS-201B7), a hepatocellular carcinoma cell line (HuH-7), and a human adult hepatocyte. Normalized fluorescent intensity values range from red (high) to blue (low) coloring, and the resultant heat map is shown with gene symbols. (TIF) The combined list of both hESC-enriched genes and hepatic genes is shown in S7 Table. (TIF) [file pone.0123193.s004.tif]

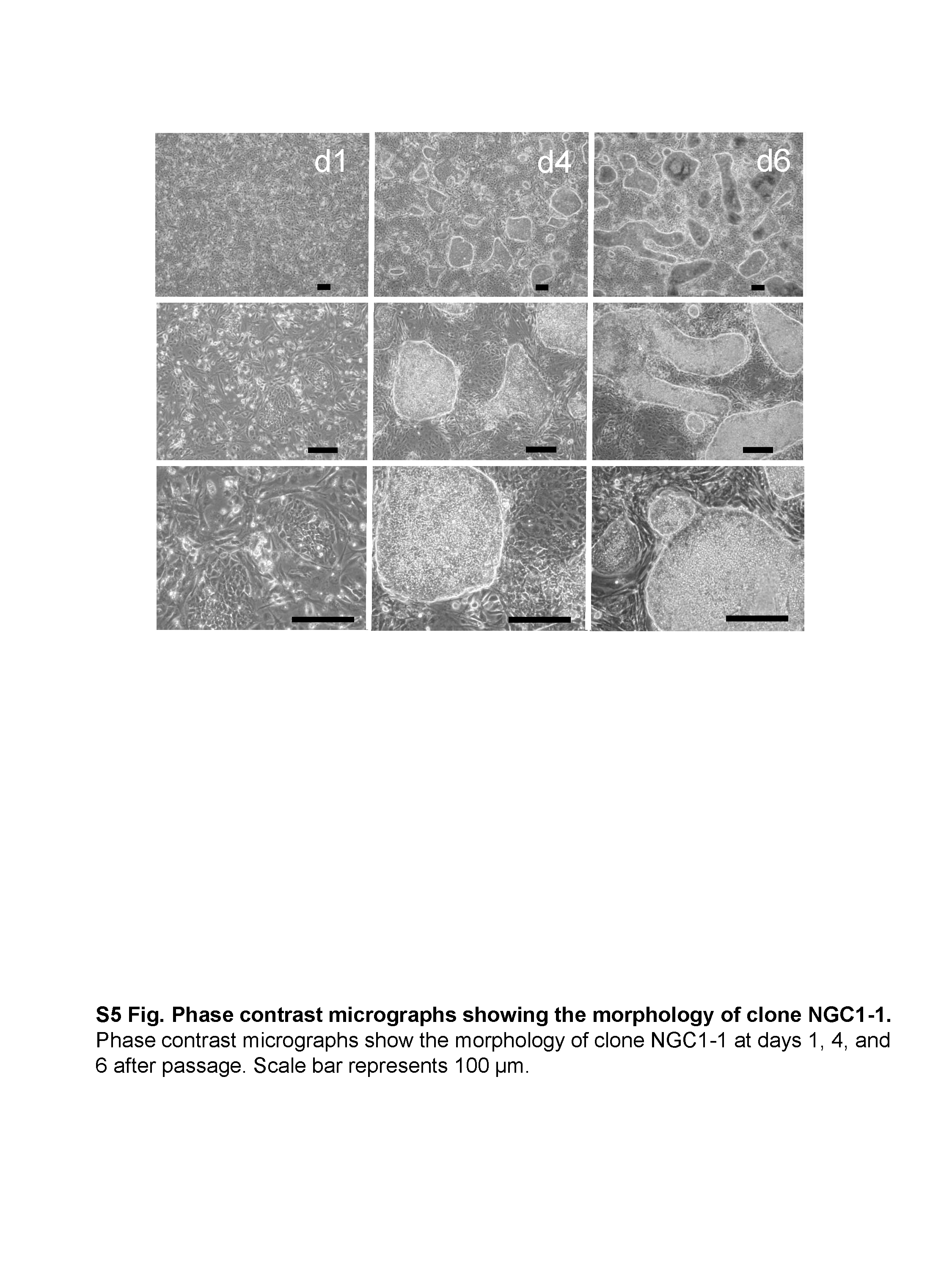

Supplement: S5 Fig — Phase contrast micrographs show the morphology of clone NGC1-1 at days 1, 4, and 6 after passage. Scale bar represents 100 μm. (TIF) (TIF) [file pone.0123193.s005.tif]

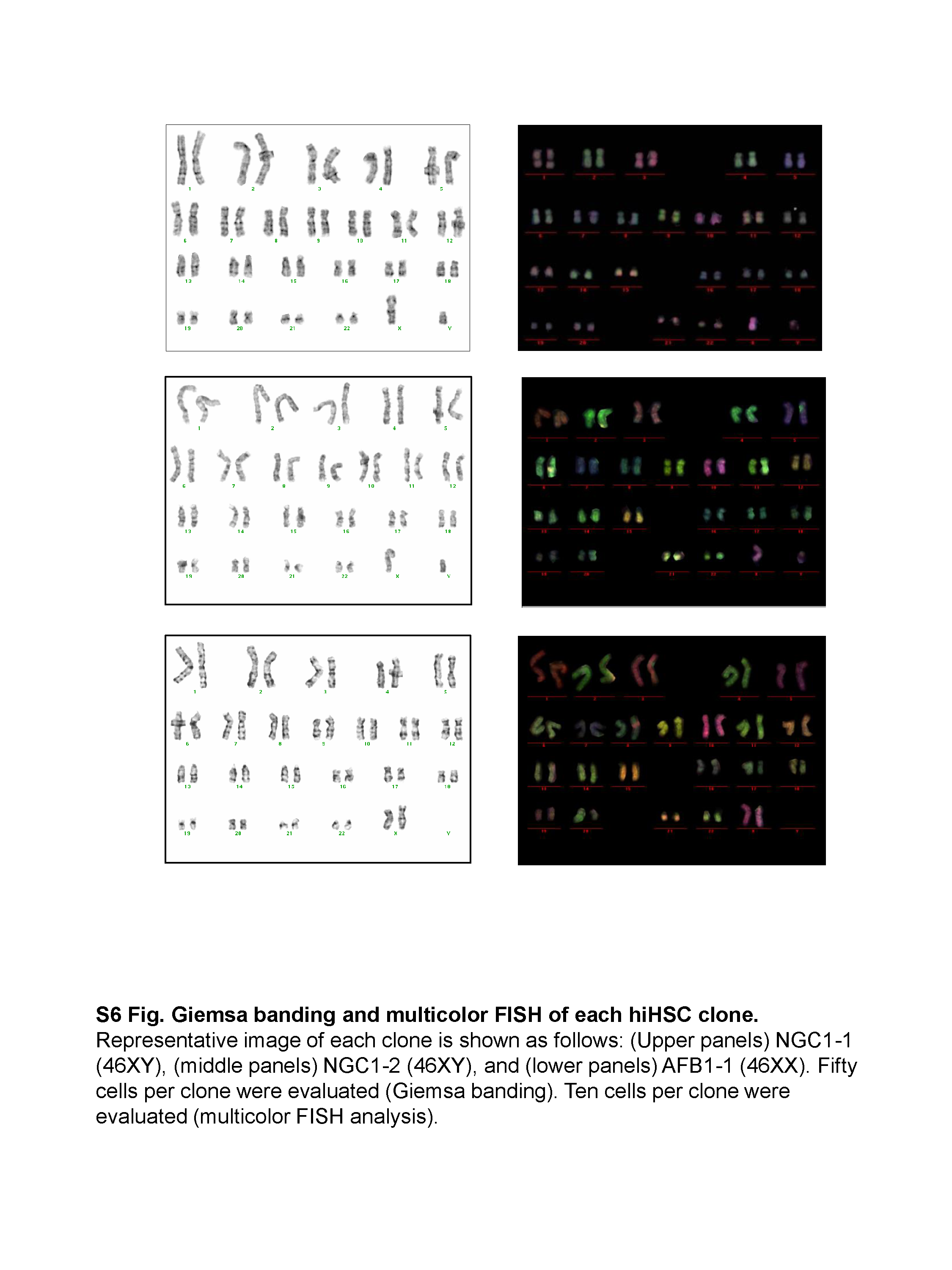

Supplement: S6 Fig — Representative image of each clone is shown as follows: (Upper panels) NGC1-1 (46XY), (middle panels) NGC1-2 (46XY), and (lower panels) AFB1-1 (46XX). Fifty cells per clone were evaluated (Giemsa banding). Ten cells per clone were evaluated (multicolor FISH analysis). (TIF) (TIF) [file pone.0123193.s006.tif]

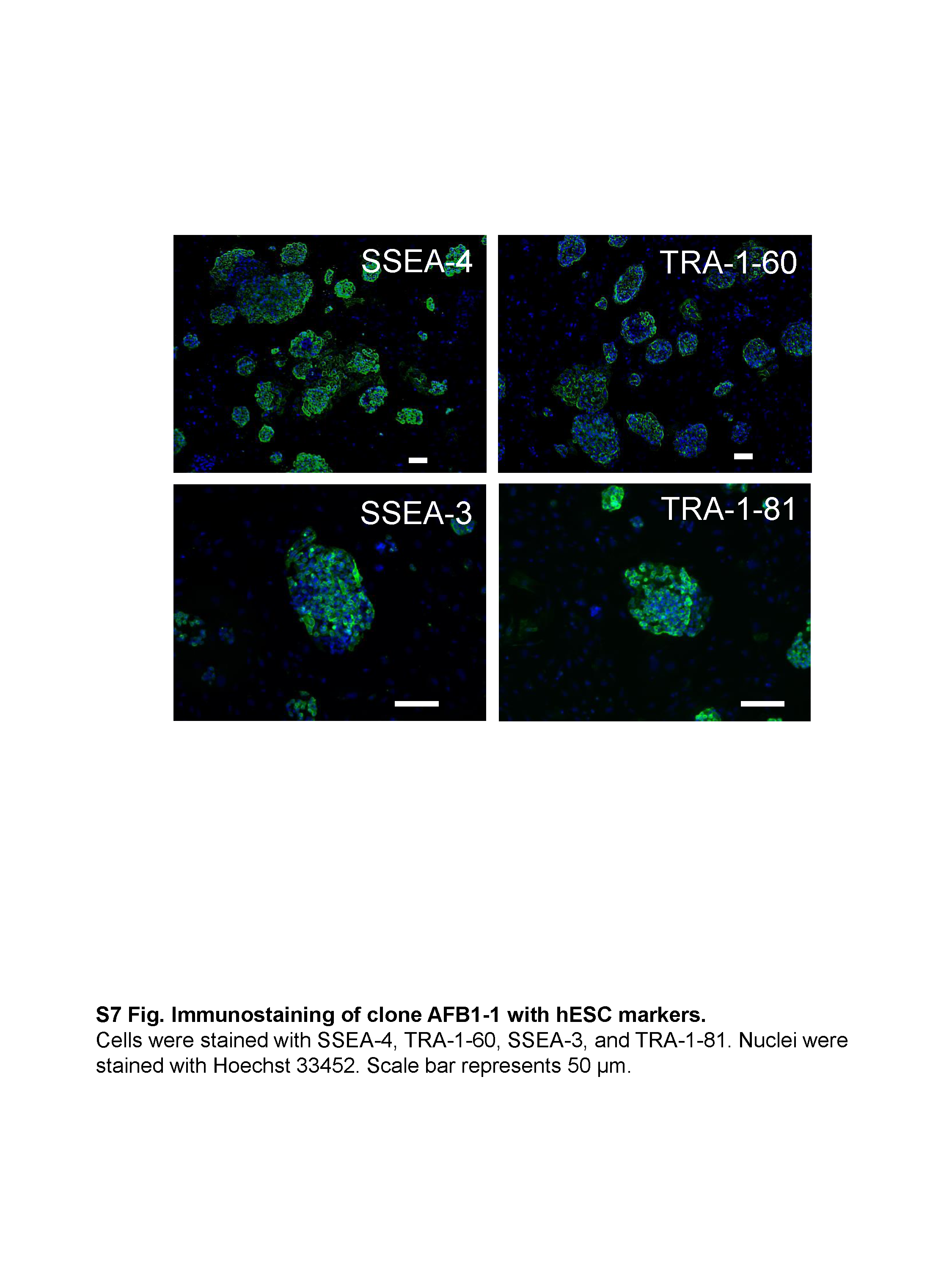

Supplement: S7 Fig — Cells were stained with SSEA-4, TRA-1-60, SSEA-3, and TRA-1-81. Nuclei were stained with Hoechst 33452. Scale bar represents 50 μm. (TIF) (TIF) [file pone.0123193.s007.tif]

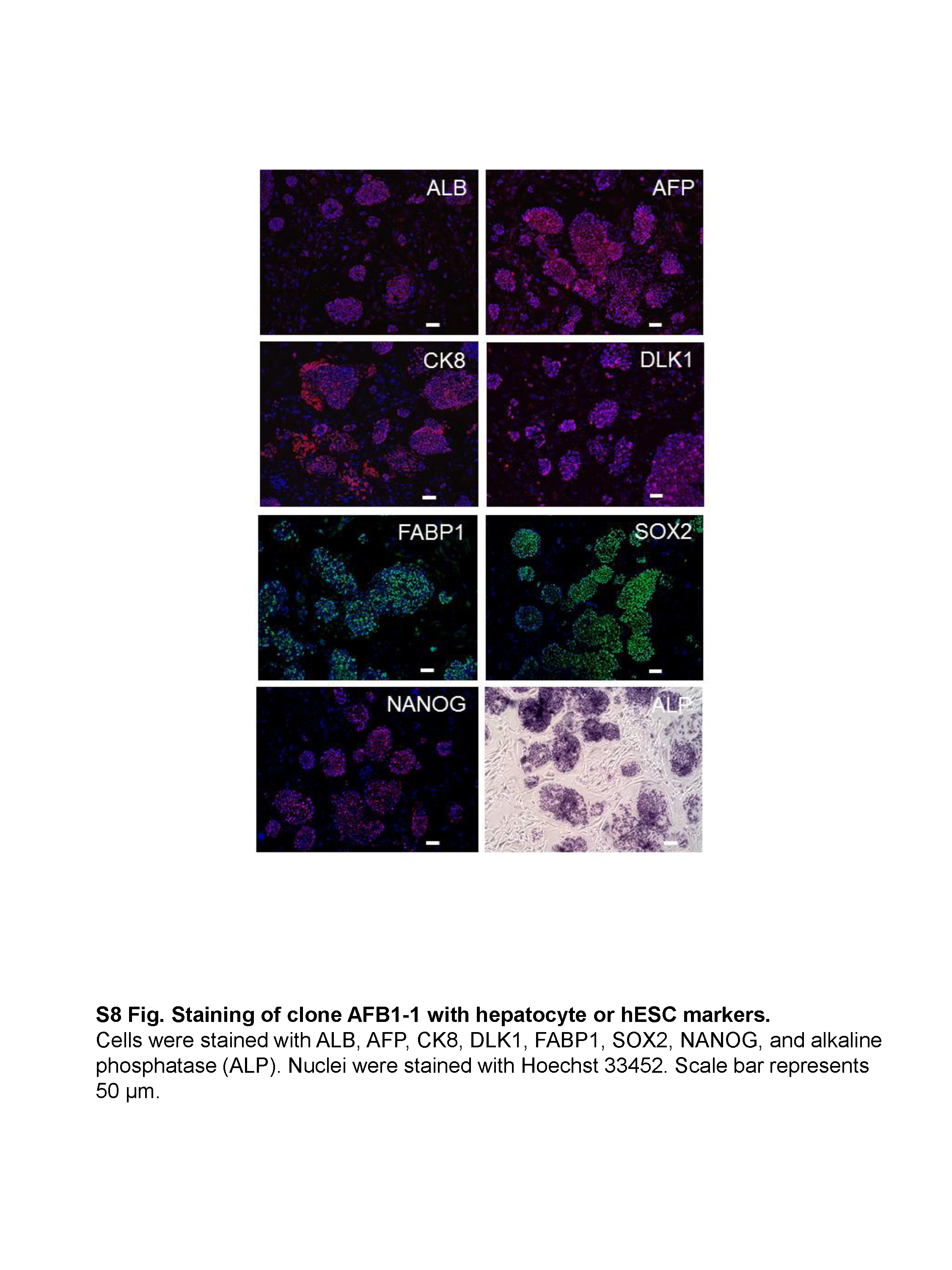

Supplement: S8 Fig — Cells were stained with ALB, AFP, CK8, DLK1, FABP1, SOX2, NANOG, and alkaline phosphatase (ALP). Nuclei were stained with Hoechst 33452. Scale bar represents 50 μm. (TIF) (TIF) [file pone.0123193.s008.tif]

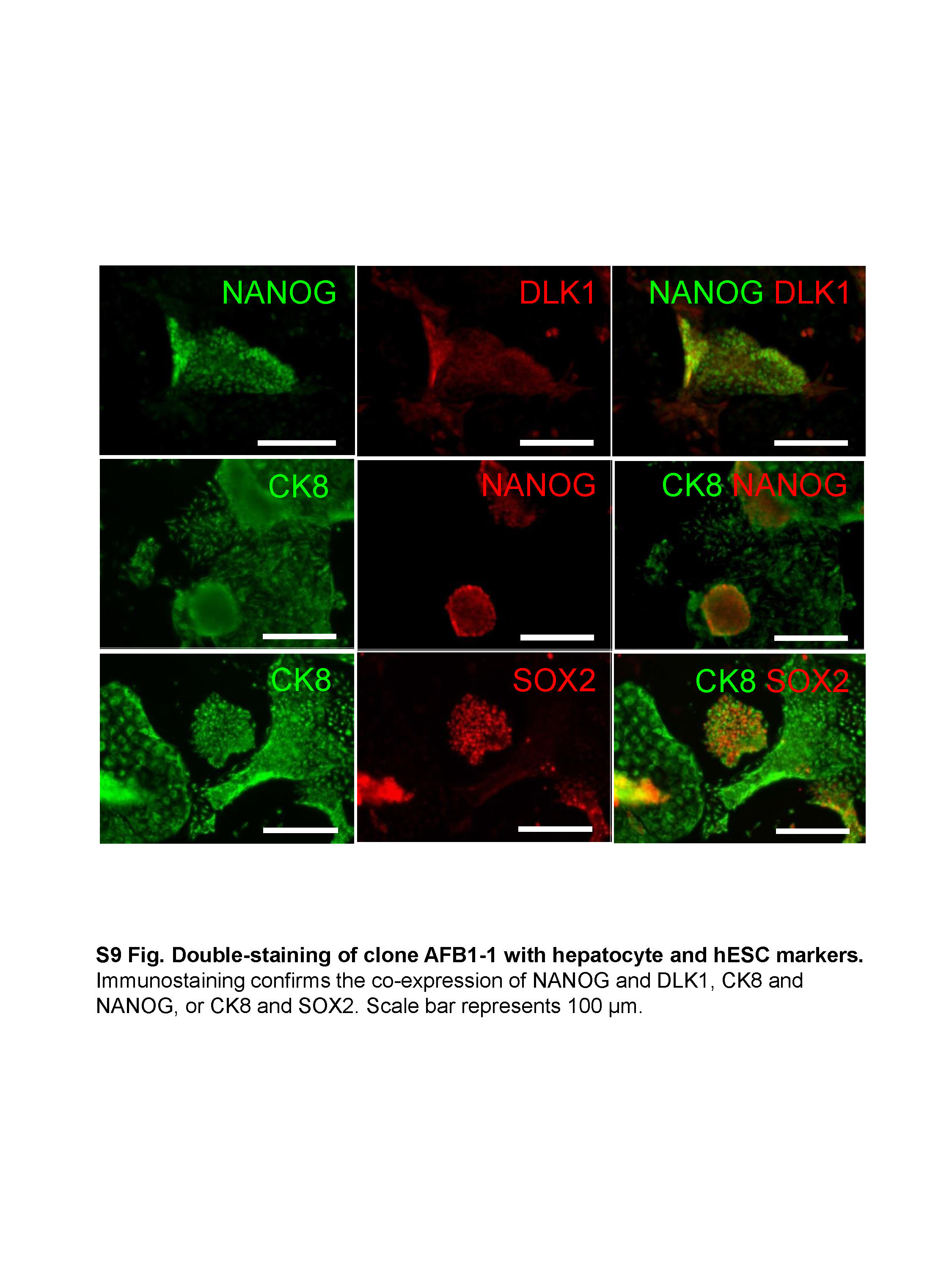

Supplement: S9 Fig — Immunostaining confirms the co-expression of NANOG and DLK1, CK8 and NANOG, or CK8 and SOX2. Scale bar represents 100 μm. (TIF) (TIF) [file pone.0123193.s009.tif]

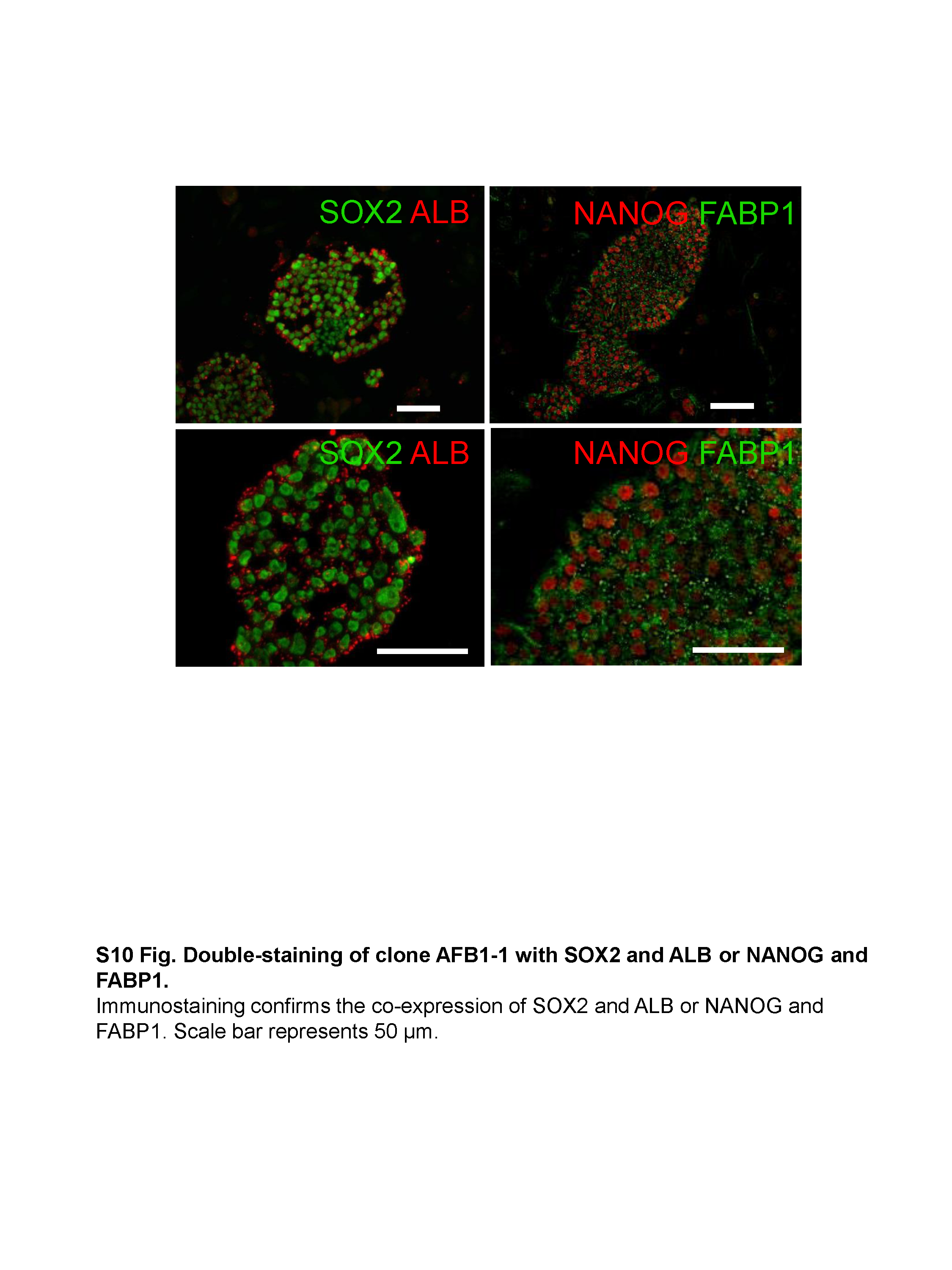

Supplement: S10 Fig — Immunostaining confirms the co-expression of SOX2 and ALB or NANOG and FABP1. Scale bar represents 50 μm. (TIF) (TIF) [file pone.0123193.s010.tif]

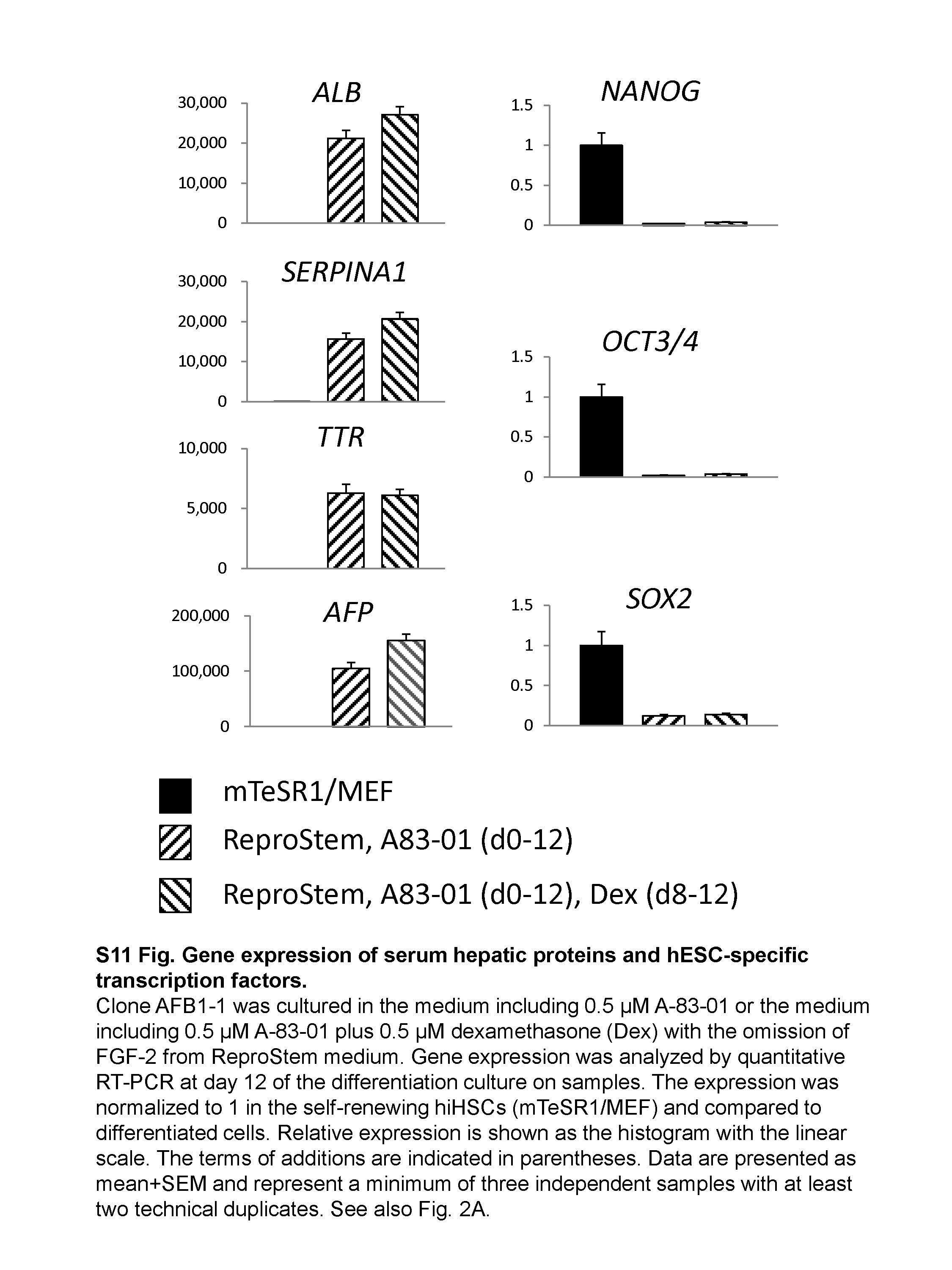

Supplement: S11 Fig — Clone AFB1-1 was cultured in the medium including 0.5 μM A-83-01 or the medium including 0.5 μM A-83-01 plus 0.5 μM dexamethasone (Dex) with the omission of FGF-2 from ReproStem medium. Gene expression was analyzed by quantitative RT-PCR at day 12 of the differentiation culture on samples. The expression was normalized to 1 in the self-renewing hiHSCs (mTeSR1/MEF) and compared to differentiated cells. Relative expression is shown as the histogram with the linear scale. The terms of additions are indicated in parentheses. Data are presented as mean+SEM and represent a minimum of three independent samples with at least two technical duplicates. (TIF) See also Fig 2A. (TIF) [file pone.0123193.s011.tif]

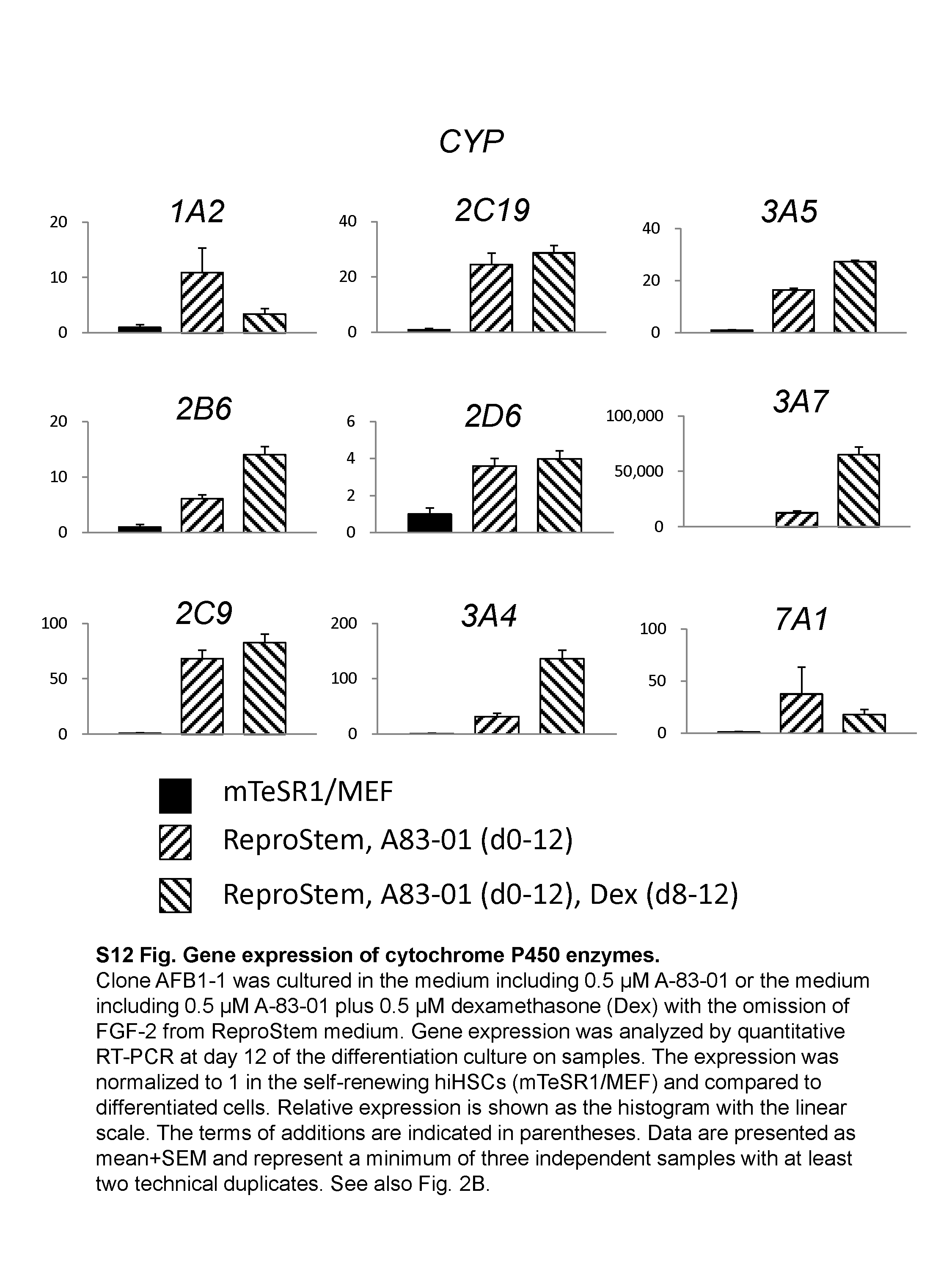

Supplement: S12 Fig — Clone AFB1-1 was cultured in the medium including 0.5 μM A-83-01 or the medium including 0.5 μM A-83-01 plus 0.5 μM dexamethasone (Dex) with the omission of FGF-2 from ReproStem medium. Gene expression was analyzed by quantitative RT-PCR at day 12 of the differentiation culture on samples. The expression was normalized to 1 in the self-renewing hiHSCs (mTeSR1/MEF) and compared to differentiated cells. Relative expression is shown as the histogram with the linear scale. The terms of additions are indicated in parentheses. Data are presented as mean+SEM and represent a minimum of three independent samples with at least two technical duplicates. (TIF) See also Fig 2B. (TIF) [file pone.0123193.s012.tif]

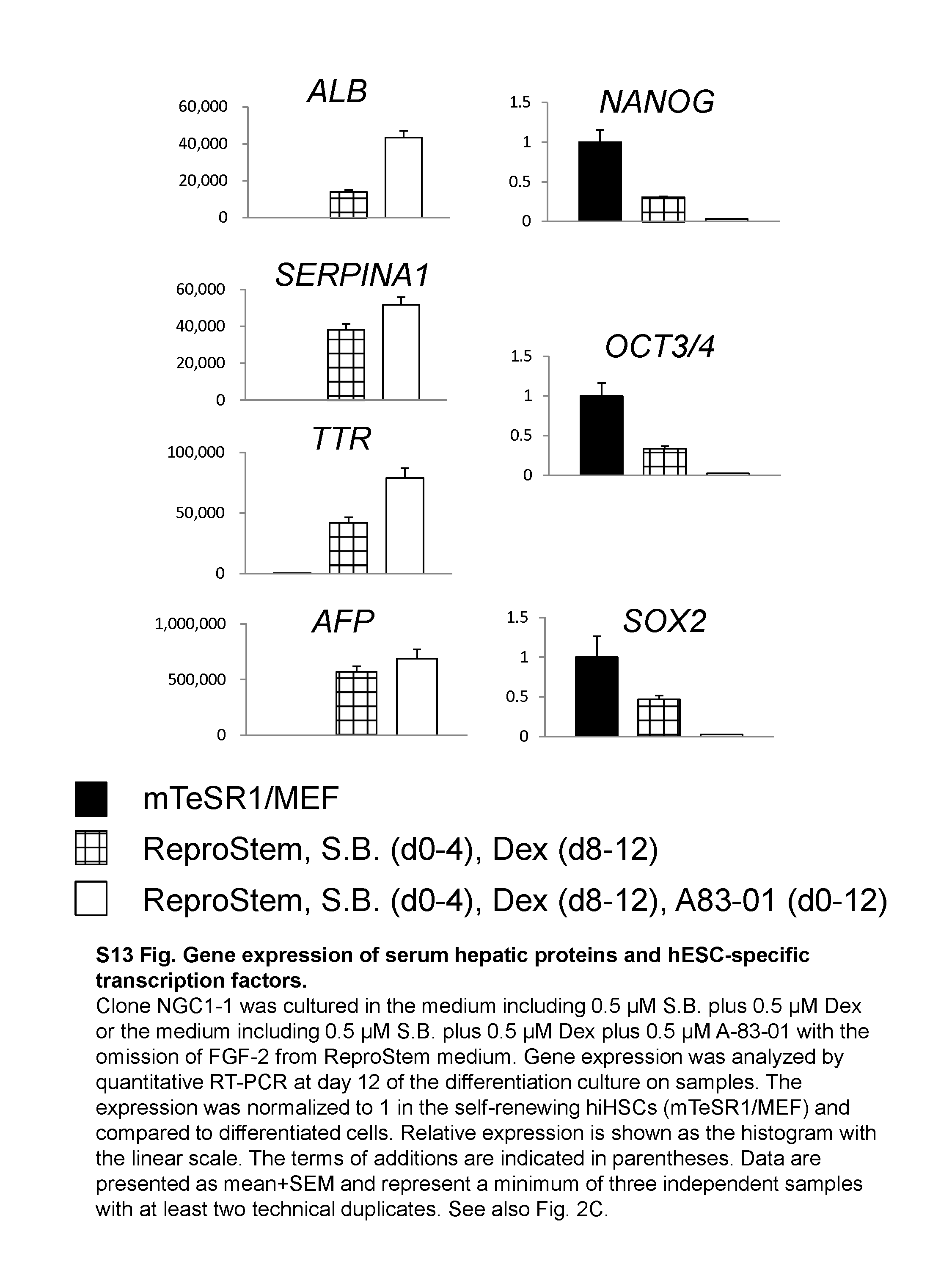

Supplement: S13 Fig — Clone NGC1-1 was cultured in the medium including 0.5 μM S.B. plus 0.5 μM Dex or the medium including 0.5 μM S.B. plus 0.5 μM Dex plus 0.5 μM A-83-01 with the omission of FGF-2 from ReproStem medium. Gene expression was analyzed by quantitative RT-PCR at day 12 of the differentiation culture on samples. The expression was normalized to 1 in the self-renewing hiHSCs (mTeSR1/MEF) and compared to differentiated cells. Relative expression is shown as the histogram with the linear scale. The terms of additions are indicated in parentheses. Data are presented as mean+SEM and represent a minimum of three independent samples with at least two technical duplicates. (TIF) See also Fig 2C. (TIF) [file pone.0123193.s013.tif]

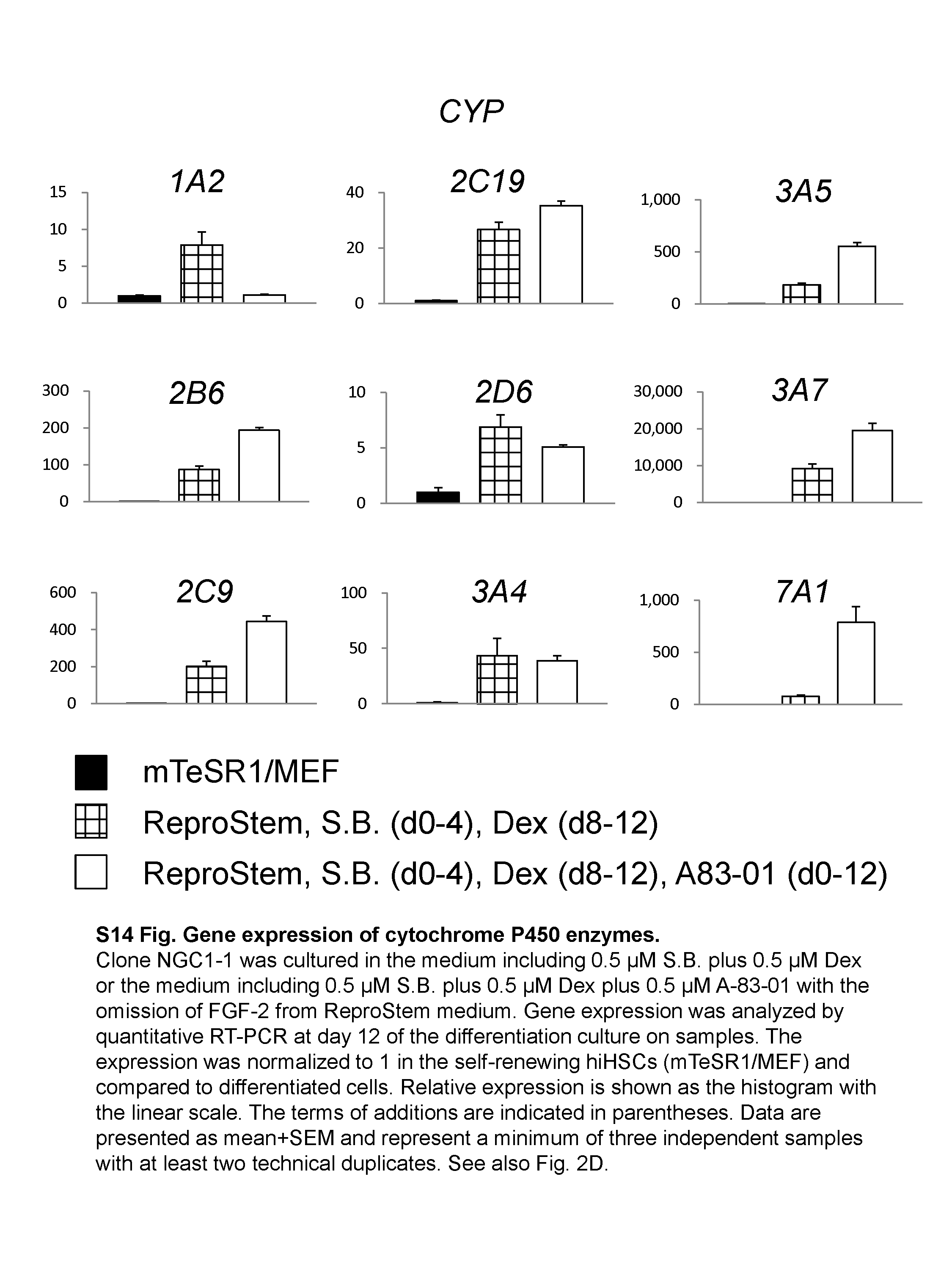

Supplement: S14 Fig — Clone NGC1-1 was cultured in the medium including 0.5 μM S.B. plus 0.5 μM Dex or the medium including 0.5 μM S.B. plus 0.5 μM Dex plus 0.5 μM A-83-01 with the omission of FGF-2 from ReproStem medium. Gene expression was analyzed by quantitative RT-PCR at day 12 of the differentiation culture on samples. The expression was normalized to 1 in the self-renewing hiHSCs (mTeSR1/MEF) and compared to differentiated cells. Relative expression is shown as the histogram with the linear scale. The terms of additions are indicated in parentheses. Data are presented as mean+SEM and represent a minimum of three independent samples with at least two technical duplicates. (TIF) See also Fig 2D. (TIF) [file pone.0123193.s014.tif]

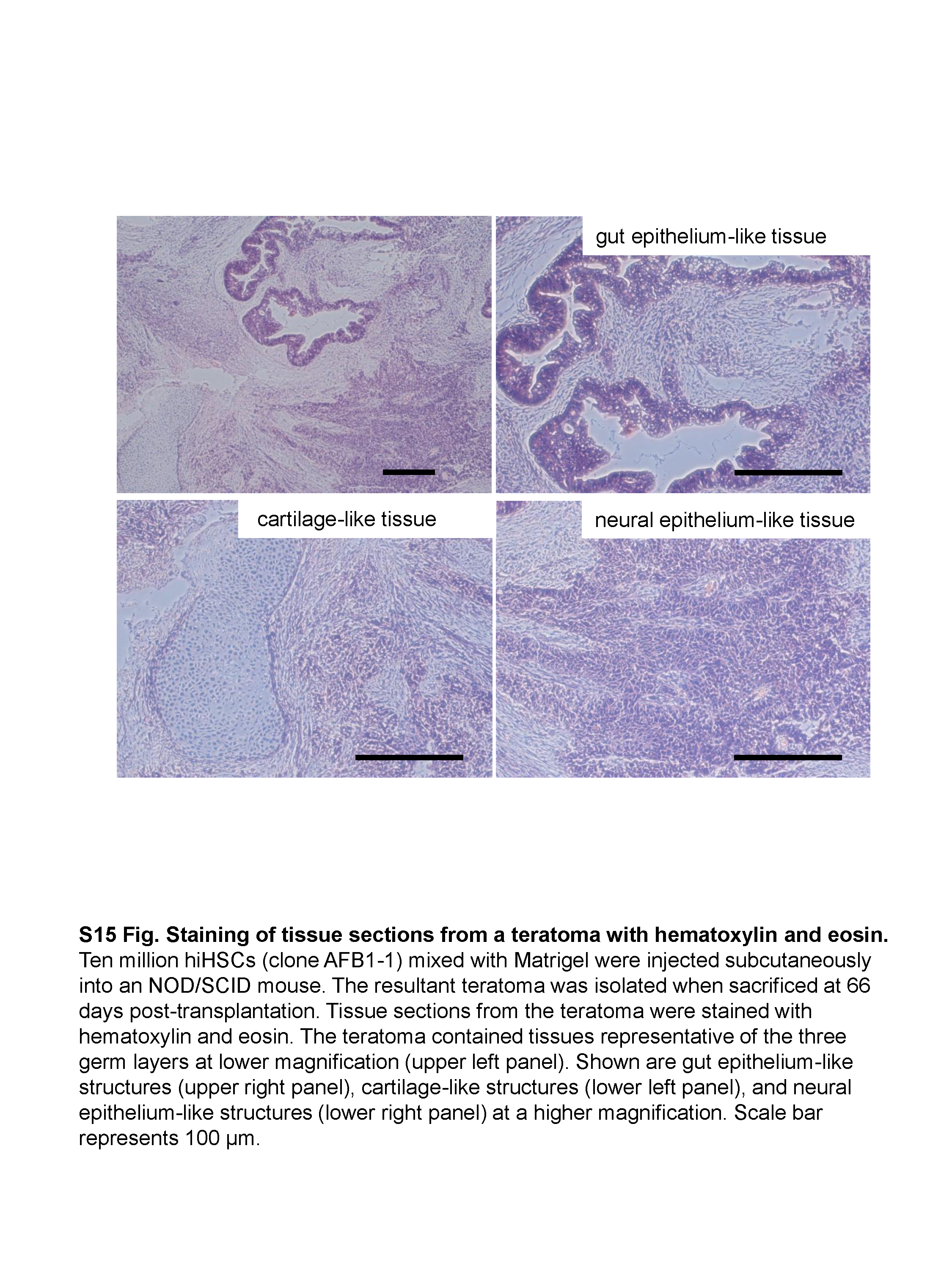

Supplement: S15 Fig — Ten million hiHSCs (clone AFB1-1) mixed with Matrigel were injected subcutaneously into an NOD/SCID mouse. The resultant teratoma was isolated when sacrificed at 66 days post-transplantation. Tissue sections from the teratoma were stained with hematoxylin and eosin. The teratoma contained tissues representative of the three germ layers at lower magnification (upper left panel). Shown are gut epithelium-like structures (upper right panel), cartilage-like structures (lower left panel), and neural epithelium-like structures (lower right panel) at a higher magnification. Scale bar represents 100 μm. (TIF) (TIF) [file pone.0123193.s015.tif]
